# Broadening the semiaquatic scene: Quantification of long bone microanatomy across pinnipeds

**DOI:** 10.1002/ar.70058

**Published:** 2025-10-13

**Authors:** Apolline Alfsen, Christian de Muizon, Olivier Lambert, Giovanni Bianucci, Antonia R. Kaffler, Matthew R. McCurry, Alexandra Houssaye, Rafael M. Varas‐Malca, Rodolfo Salas‐Gismondi, Oliver Hampe, Eli Amson

**Affiliations:** ^1^ Evolutionary Morphology Museum für Naturkunde – Leibniz‐Institut für Evolutions‐ und Biodiversitätsforschung Berlin Germany; ^2^ Freie Universität Berlin, Fachbereich Geowissenschaften Institut für Geologische Wissenschaften, Fachrichtung Paläontologie Berlin Germany; ^3^ Paleontology Department State Museum of Natural History Stuttgart Stuttgart Germany; ^4^ CR2P (UMR 7207 CNRS‐MNHN‐Sorbonne Université), Origins and Evolution Department Muséum national d'Histoire naturelle MNHN Paris France; ^5^ D.O. Terre et Histoire de la Vie Institut royal des Sciences naturelles de Belgique Brussels Belgium; ^6^ Dipartimento di Scienze della Terra University of Pisa Pisa Italy; ^7^ Australian Museum Research Institute Sydney New South Wales Australia; ^8^ Earth and Sustainability Science Research Centre, School of Biological, Earth and Environmental Sciences University of New South Wales Sydney Australia; ^9^ UMR 7179 MECADEV CNRS‐MNHN Paris France; ^10^ Departamento de Paleontología de Vertebrados Museo de Historia Natural, Universidad Nacional Mayor de San Marcos Lima Peru

**Keywords:** bone microanatomy, Mustelidae, Pinnipedia, semiaquatic locomotion, weight‐bearing

## Abstract

Investigations of bone microanatomy are commonly used to explore lifestyle strategies in vertebrates. While distinct microanatomical limb bone features have been established for exclusively aquatic and terrestrial lifestyles, identifying clear patterns for the semiaquatic lifestyle remains more challenging. Pinnipeds and mustelids provide an ideal framework for studying the diversity of semiaquatic adaptations. Here, we tested whether their humerus and femur showed significant differences in microanatomical and biomechanical parameters along the diaphysis relative to locomotor strategies. We μCT‐scanned extant pinnipeds and included semiaquatic and generalist mustelids to build a reference for the amphibious lifestyle. Statistical analyses showed that comparisons away from the growth center distinguished species with non‐weight‐bearing hind limbs from those with all‐limb weight‐bearing. Among the latter, otariids and semiaquatic mustelids shared highly compact bones and a reduced medullary cavity, though otariids exhibited a more gradual medullocortical transition with more trabeculae. Contrary to the prevailing assumption, our findings indicate bone mass increase can also be associated with fast‐swimming predators diving beyond shallow depths. Phocids, which cannot bear weight on their hind limbs, include species that spend 70%–80% of their lives at sea. These species exhibited extremely spongious bones: low compactness, thin cortex, and a trabeculae‐filled medullary region–reminiscent of the osteoporotic‐like condition seen in some extant cetaceans. Additionally, the shared humerus–femur pattern, regardless of locomotor type, suggests that skeletal adaptations can be systemic in carnivorans. This new comparative dataset broadens the spectrum of microanatomical patterns associated with an amphibious lifestyle and provides a foundation for better deciphering extinct species' locomotion.

## 1 INTRODUCTION

Bone microstructure has been widely studied to understand its relationship with locomotion, with most studies focusing on single‐slice analyses at the midshaft or growth center of long bones (Amson & Bibi, 2021; Amson & Kilbourne, 2019; Bader et al., 2022; Canoville & Laurin, 2009, 2010; Buffrénil, Muizon, et al., 2021; Buffrénil, Ricqlès, et al., 2021; Germain & Laurin, 2005; Gônet et al., 2023; Houssaye & Botton‐Divet, 2018; Kriloff et al., 2008; Nakajima & Endo, 2013; Nieminen et al., 2024; Quemeneur et al., 2013; Straehl et al., 2013). Two distinct patterns have been described in association with an aquatic lifestyle. The first is observed in shallow‐diving, relatively slow‐swimming taxa, such as sirenians, which exhibit high bone compactness, characterized by a thick cortex and a reduced medullary cavity (Canoville & Laurin, 2009; Houssaye, 2009; Kriloff et al., 2008; Nakajima & Endo, 2013). This high compactness provides negative buoyancy to counteract the positive buoyancy of air‐filled lungs, as well as a natural ballast effect to facilitate underwater locomotion. In contrast, deep‐diving species tend to exhibit spongious bones characterized by a thin cortex and a medullary region filled with trabeculae (Fish & Stein, 1991; Gray et al., 2007; Houssaye et al., 2016; Stein, 1989; Wall, 1983). While these traits are commonly associated with active swimming and dynamic buoyancy regulation, recent studies suggest that deep divers do not uniformly display osteoporotic bones, at least in their ribs (Canoville et al., 2016; Houssaye et al., 2016). Although the osteoporotic pattern is documented in fully aquatic taxa—clearly distinguishing them from their terrestrial counterparts—the microanatomical patterns of semiaquatic species remain less clear (Laurin et al., 2011). Carnivora provides the perfect framework for studying these amphibious adaptations. Pinnipeds and mustelids, in particular, exhibit a diversity of amphibious specializations, with distinct swimming types and terrestrial locomotion strategies (Tarasoff et al., 1972).

**FIGURE 1:**
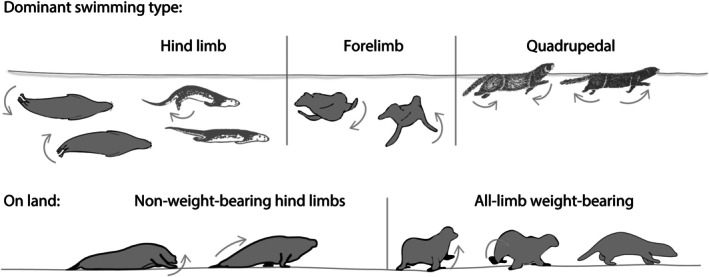
Locomotor categories used in this analysis. Modified from Esteban et al. (2023), Fish (2001), and Lodé (1999).

Mustelids and pinnipeds show notable differences in weight‐bearing on land. All mustelids can support their weight on all four limbs, and in water, they swim either using all their limbs (alternate paddling) or by generating a more powerful propulsion driven by their hind limbs and tail (Fish, 1994; Lodé, 1999). Pinnipeds, however, display more extreme variations in their terrestrial locomotion. Otariids (fur seals and sea lions) bear their weight on all four limbs, whereas phocids (true seals) keep most of their body in contact with the ground, unable to stand on their hind limbs (Berta, 2018; Kuhn & Frey, 2012). At sea, otariids rely on a forelimb‐dominated propulsion (Jeanniard‐du‐Dot & Guinet, 2021), whereas most of the propulsion in phocids is performed by the hind limbs. On land, phocids rely on axial undulation, with non‐weight‐bearing hind limbs and limited support from the forelimbs (Berta et al., 2022; Figure 1; Table 1). On land, walruses (Odobenidae) walk similarly to otariids with all‐limb weight‐bearing; in the water, they move their body laterally like phocids but also use their forelimbs, as otariids do, for vertical paddling (King, 1983). All mustelids and pinnipeds retain some dependency on the terrestrial environment, though the nature and degree of this dependency vary widely between and within the two clades. Semiaquatic mustelids, with the exception of the sea otter (*Enhydra lutris*), tend to return daily onshore for resting and grooming (Flaherty, 2022), whereas pinnipeds show greater variation in their dependency on the terrestrial environment.

**TABLE 1:** Locomotor category of each clade sampled; category 6, only comprising one species, was excluded from statistical analyses.

First level of category	Mustelidae		Pinnipedia			
	1	2	3	4	5	6
Clade	Generalist Mustelidae	Semiaquatic Mustelidae	Otariidae	Monachinae	Phocinae	Odobenidae
Dominant swimming style	Quadrupedal swimmers	Hind limb swimmers	Forelimb swimmers	Hind‐Forelimb swimmers	Hind limb swimmers	Intermediate swimming style
Weight bearing limb on land = second level of category	All‐limb weight‐bearing	All‐limb weight‐bearing	All‐limb weight‐bearing	Non‐weight‐bearing hind limbs	Non‐weight‐bearing hind limbs	All‐limb weight‐bearing

Pinnipeds differ from birth in their amphibious strategy. Otariid pups nurse on land for an extended period before following their mother at sea (Sepúlveda & Harcourt, 2021), whereas some phocid pups can dive within hours of birth (Carter et al., 2017; Costa & Maresh, 2022). Adult otariids spend on average ~50% of their time in the water, making daily excursions for foraging at sea and returning on land to rest (Beentjes, 1989; Costa & Valenzuela‐Toro, 2021; Horning & Trillmich, 1997). Sea lions and fur seals have different feeding grounds. Sea lions forage nearshore with shorter trips at sea than fur seals, which can make longer trips farther from haul‐out sites (Costa & Valenzuela‐Toro, 2021). Phocids, in contrast, exhibit higher aquatic dependency. While time spent at sea by adults is not known for all species, monitored species were found to stay in the water between 60% and 80% of their life (Adam, 2005; Costa & Valenzuela‐Toro, 2021; Horning & Trillmich, 1997; Krause et al., 2016). Similarly, adult walruses are known to spend between 60% and 85% of their time at sea (Udevitz et al., 2009).

Given the contrasting weight‐bearing strategies and varying aquatic dependencies within pinnipeds and mustelids, investigating these clades can let us identify microanatomical adaptations reflecting the aquatic locomotion and the influence of weight‐bearing on specific limbs. Previous studies have highlighted challenges in differentiating terrestrial and amphibious taxa using single‐slice compactness analyses of long bones (Canoville & Laurin, 2009; Germain & Laurin, 2005; Laurin et al., 2004). Some researchers have also noted a shared pattern of higher compactness and increased resistance ratios among fossorial and semiaquatic species (Geiger et al., 2013; Montoya‐Sanhueza & Chinsamy, 2017; Parsi‐Pour & Kilbourne, 2020). Additionally, in long bones, the growth center does not systematically align with the midshaft (Buffrénil & Quilhac, 2021; Nakajima et al., 2014), stressing the need for more comprehensive analyses. To address these limitations, we used micro‐Computed Tomography (CT) scanning and quantified a combination of microanatomical parameters with longitudinal profiling along the complete bone shaft to distinguish semiaquatic clades within Carnivora. Such analyses along the complete shaft of long bones have been done before, although looking at slightly different microanatomical parameters (e.g., Kilbourne & Hutchinson, 2019; Parsi‐Pour & Kilbourne, 2020). We quantified the humeral and femoral microanatomy of 38 species of generalist and semiaquatic mustelids and pinnipeds. The distribution of bone tissue across the cortex and medullary region, as well as cross‐sectional shape, was analyzed by calculating the mean value of each parameter along the entire bone diaphysis and by generating proximodistal profiles. We used two levels of functional categories. With the first one, we independently compared each of the locomotor strategies employed here (these overlap with the clades within pinnipeds). We then defined second‐level categories to compare species with all‐limb weight‐bearing against species with a non‐weight‐bearing hind limb.

The main objective of this study is to capture and characterize microanatomical variations in long bones among semiaquatic carnivorans, particularly pinnipeds, for whom this aspect remains understudied. Since both internal and external bone morphology is shaped by mechanical loading, we expect the humeral and femoral internal structure to reflect the contrast between all‐limb weight‐bearing and non‐weight‐bearing hind limb taxa. Bones involved in weight‐bearing are predicted to have a bulkier shape and to exhibit a higher compactness than non‐weight‐bearing bones, reflecting adaptations to increased axial loading. We anticipate that all‐limb weight‐bearing taxa will show greater resistance to axial compression than to bending in the femur, compared to non‐weight‐bearing hind limb taxa. Additionally, we expect the strain of swimming to differently affect the humerus and femur depending on their roles in the animal's aquatic propulsion, resulting in distinct patterns of resistance to bending and axial compression. The results of this study should be of particular interest for reconstructions of extinct species' locomotion, particularly in the context of early cetaceans, for whom well‐preserved fossils have been found.

## 2 MATERIALS AND METHODS

### 2.1 Data acquisition

To perform a comparative analysis of the humerus and femur bone microanatomy across extant pinnipeds, we sampled one humerus and one femur from 61 specimens (21 species) representing ca. 70% of Otariidae, 50% of Phocidae, and including the only extant representative of Odobenidae (*Odobenus rosmarus*). The sample was completed with one humerus and one femur from 26 specimens (17 species) of extant mustelids representing eight semiaquatic species and nine generalist species. Specimens were loaned from multiple institutions (Tables S1 and S2) for direct measurements and μCT‐scanning on site or at the Museum für Naturkunde, Berlin, with a Phoenix|X‐ray Nanotom (GE Sensing and Inspection Technologies GmbH, Wunstorf, Germany) and a FF85‐CT‐System (YXLON GmbH, Hamburg, Germany); most CT scans of mustelid specimens were downloaded from Morphosource (Project ID: 00000C674 from Kilbourne & Hutchinson, 2019; Parsi‐Pour & Kilbourne, 2020). Wild adult individuals were selected based on their fused epiphyses to ensure skeletal maturity, as bone microstructure is subject to strong variability during earlier stages of growth. Bones from the right side were preferentially selected when available to standardize sampling and reduce variability. As most collection individuals of these taxa have no sex identification, it was not possible to restrict the sampling to only one identified sex. To control intraspecific variation, we ran a PCA and compared the distribution of our sample by individuals (sex was known in some cases) and by species (using species mean across individuals) (Figure S1).

Functional bone length was measured directly on each bone using an electronic digital Kraftixx caliper, kwb Germany, Stuhr, except for six specimens that were accessed only digitally (CT scans) and were measured on Dragonfly 3D World (2024‐Windows). All bones were virtually oriented in a standard position in Dragonfly: *X*, *Y*, and *Z* axes, respectively aligned with mediolateral, anteroposterior, and proximodistal axes; the bone was set in a vertical position by aligning the center of the proximal and distal metaphyses along the *Z* axis (using the software's *Y* and *X* views); on the *Z* view, the bone was positioned with its anterior side facing up and this proximo‐distal view was exported as a TIFF stack. Microanatomical parameters were acquired using the BoneJ plugin in Fiji (Doube et al., 2010; Schneider et al., 2012) on complete TIFF stacks and in R with the BoneProfileR package (Gônet et al., 2021) on a subsample of TIFFs. A Fiji macro from Amson (2019) was adapted and run on the complete TIFF stack of each specimen to measure global compactness (Cg, %), cross‐sectional area (CSA, mm^2^), total area (mm^2^), second moment of area on the cranio‐caudal axis (SMA_CC_, mm^4^) and on the medio‐lateral axis (SMA_ML_, mm^4^) and cross‐sectional perimeter (mm); from the total area and CSA, we calculated total diaphyseal volume (TV, mm^3^), resistance ratios on cranio‐caudal *R*_CC_ (SMA_CC_/CSA) and medio‐lateral axis *R*_ML_ (SMA_ML_/CSA), and a bulkiness index (BI: minimal diaphyseal perimeter divided by bone total length). CSA represents the bone's ability to resist axial compression; a higher CSA indicates greater resistance to compressive forces along the bone's longitudinal axis. SMA characterizes resistance to bending along a specific axis. Specifically, SMA_CC_ corresponds to bending resistance in the medio‐lateral (ML) plane, while SMA_ML_ reflects resistance in the cranio‐caudal (CC) plane (Kilbourne & Hutchinson, 2019; Figure 1). In both cases, higher SMA values indicate increased structural resistance to bending in the respective direction. Resistance ratios were used to compare bone resistance to axial compression versus resistance to bending about a given axis (*R*_CC_ and *R*_ML_ for each corresponding axis). The bulkiness index assessed the ability to resist bending and shearing stresses; a high index indicates a short and large bone (Dewaele et al., 2019). At the end of each Fiji acquisition, artefactual peaks in compactness and in perimeter helped to eliminate problematic slices with a discontinuous cortex (more frequent in very spongious bones), following Amson (2019). This macro also let us label four key positions using bone functional length (BL; i.e., for femur and humerus: the proximo‐distal maximal distance between the articular head and the distal condyle). Due to the single slice analysis process of the BoneProfileR package, we had to restrict the number of slices analyzed per specimen. Five slices were found to be sufficient to capture the diaphyseal variation: at 25%, 50%, 69%, and 75% of BL and an additional fifth position at the growth center (identified manually as the slice where the nutrient canal enters the medullary cavity and corresponding to the most compact slice). A preliminary examination of diaphyseal profiles showed that some species had a decrease in compactness in bones' distal half, around 69% of their length. This position was added in the slice analysis to capture that variation in the sample. With BoneProfileR, a radial analysis of 60 sectors for each of the five bone cross‐sections was completed to measure mean values of *P* and *S*. Parameter *P* stands for the distance of the medullocortical transition from the center of the slice, that is, for tubular bones the higher the *P*, the wider the medullary region (hence, the thinner the cortex, Gônet et al., 2021). Parameter *S* is proportional to the reciprocal of the slope of compactness at the medullocortical transition; a low *S* indicates an abrupt transition while a high *S* shows a gradual transition with the presence of porosity/trabecular bone in the medullary region (Gônet et al., 2021). From the results, we subsampled 100 equally spaced positions between 25% and 75% of BL for all the Fiji parameters listed above and used the five representative slices for BoneProfileR parameters. For each micro‐anatomical parameter, we devised two datasets: (A) one containing the mean value of each parameter calculated along the entire diaphysis for each species, and (B) another comprising the 100 or 5 values distributed along the diaphysis, used to examine proximodistal profiles for each species. All raw data are available in Tables S1–S7.

### 2.2 Locomotor categories

We defined five locomotor categories that reflect which limbs are involved for aquatic propulsion and which limbs are able to bear the weight of the animal on land. Categories are as follows: (1) quadrupedal paddling swimmers with all‐limb weight‐bearing; (2) hind limb dominant swimmers with all‐limb weight‐bearing; (3) forelimb dominant swimmers with all‐limb weight‐bearing; (4) hind limb dominant swimmers with intermediate forelimb implication for swimming and non‐weight‐bearing hind limbs; (5) hind limb dominant swimmers with minor forelimb implication in swimming and non‐weight‐bearing hind limbs (Figure 1 and Table 1).

**FIGURE 2:**
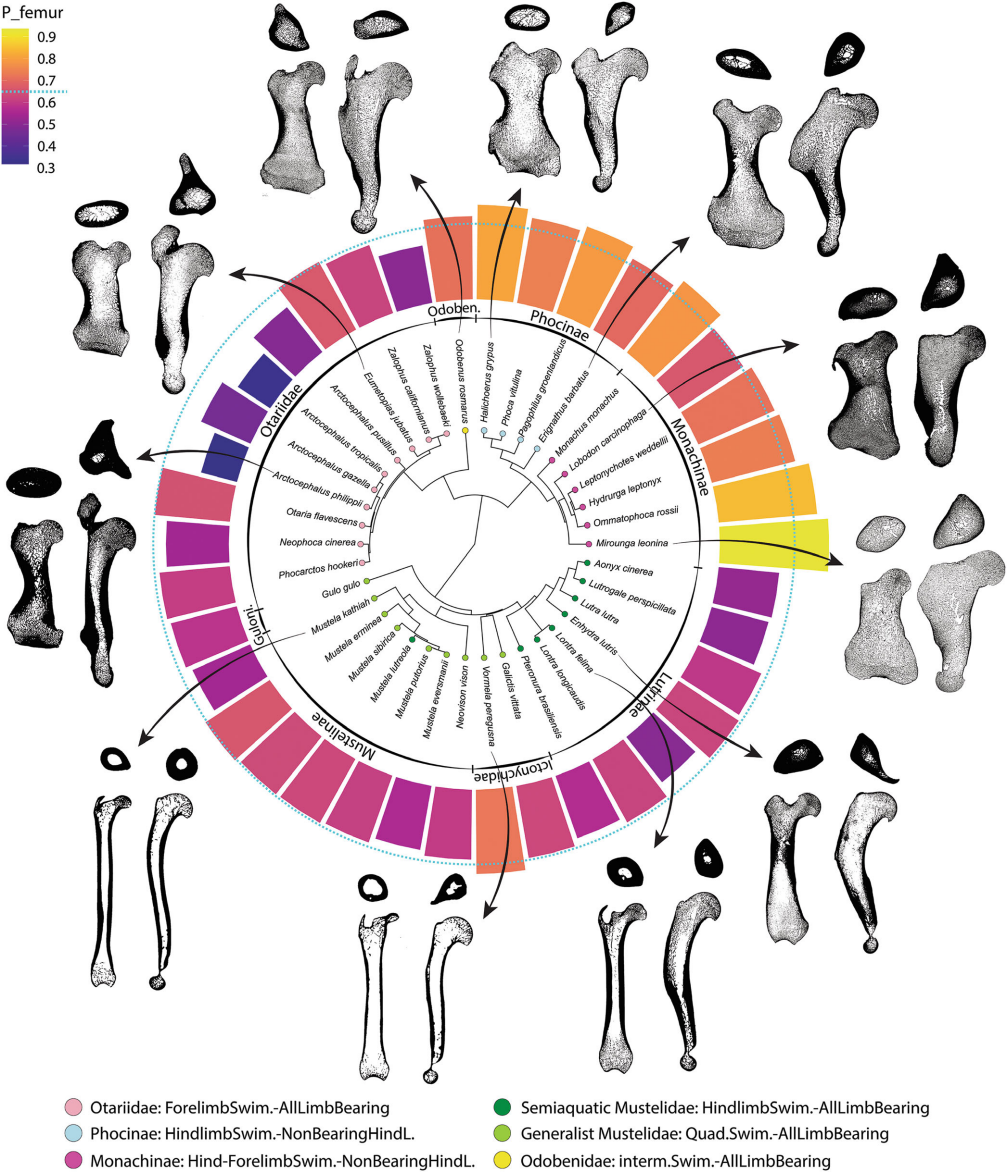
Distribution of the microanatomical parameter *P* (relative size of the medullary region) across femora of sampled carnivorans. Two examples per category (lowest and highest values of *P*) are shown in longitudinal sections with a cross‐section for each bone taken at its growth center (left: femur, right: humerus; not to scale). The dashed blue line indicates the 65% level of *P*, above which all non‐weight‐bearing species (phocids) are found.

For pinnipeds, each clade overlaps completely with a locomotor strategy, otariids representing category 3 and phocids categories 4 and 5 (Berta et al., 2018). In phocids, category 4 corresponds to monachines and 5 to phocines (Hocking et al., 2021; Ray, 1963). Monachine seals have recently been recognized as having a forelimb morphology somewhat convergent to that of otariids (Hocking et al., 2021). Both monachines and phocines seals are known to use their forelimbs for accessory propulsive movements during an otherwise primarily hind limb‐driven stroke (Muizon, 1981; Hocking et al., 2021). However, convergent forelimb morphology with otariids has, so far, only been demonstrated in monachine seals (Hocking et al., 2021). By assigning the two phocid subfamilies to two distinct locomotor categories, we tested whether the morphological convergence toward an otariid‐like forelimb is reflected in the humeral microanatomy of monachines. As locomotor strategies and clades do not overlap perfectly in mustelids, we categorized them as generalist (category 1) and semiaquatic (category 2) (tab. 1 of Kilbourne & Hutchinson, 2019; Figure 2). Generalist mustelids represent here the only category using predominantly the terrestrial environment, without a specialized locomotor habit and all limbs bearing weight on land. In the water, generalist mustelids rely on an alternate quadrupedal paddling while semiaquatic mustelids use a dorsoventral propulsive undulation with synchronized movement of their hind limbs and tail (Fish, 1994; Flaherty, 2022; Lodé, 1999). Phocids also use an undulatory movement for propulsion but driven primarily by mediolateral motions of the pelvis. While this plays an important role in aquatic locomotion, it falls outside the scope of our analysis, which focuses on long bones, and is therefore not addressed further in this study. For readability, we will refer to the first‐level categories by their clade names. We used first‐level categories, that is, each of the five categories, to compare each clade/locomotor strategy with one another. In the second‐level category set, we tested the influence of weight‐bearing, comparing species with all‐limb weight‐bearing (grouping categories 1, 2, and 3) against species with non‐weight‐bearing hind limbs (grouping categories 4 and 5). A sixth category includes the walrus: it has an intermediate swimming strategy, using both hind limb and forelimb for aquatic propulsion and all limbs bearing weight on land (Gordon, 1981; Ray, 1963). As there is only one representative of this category, it was excluded from statistical analyses and used for observational comments.

### 2.3 Statistical analyses

Given that locomotor categories align with phylogenetic groups, we conducted a preliminary assessment using a phylogenetically informed approach. First, in R v.4.5.0 (R Core Team, 2020), we trimmed the timetree from Upham et al. (2019) to match our sample (phytools package Revell, 2012). We assessed the group‐clade aggregation of our categories with the two‐block partial least squares function (*two.b.pls*; Adams & Collyer, 2018). This confirmed the strong aggregation of the categories, the so‐called “worst‐case” scenario (r‐PLS: 0.9511; *p*‐value < 0.0001; Adams & Collyer, 2018). To further examine the phylogenetic effect, we performed phylogenetically informed regressions (*lm.rrpp* function in RRPP package; Collyer & Adams, 2018) using a covariance matrix derived from our tree (*vcv.phylo* function, ape package; Paradis & Schliep, 2019). For each parameter, we conducted an ANOVA using this model to assess whether our locomotor categories significantly contributed to variation after accounting for phylogenetic covariance. For both sets of analyses, none of the tests over our six parameters were found to yield significant results (*p*‐value > 0.4). Biomechanical constraints on skeletal elements, including their microanatomy, have been well‐documented in mammals (Amson & Bibi, 2021; Etienne et al., 2025; Kilbourne & Hutchinson, 2019; Rahmat et al., 2020). Due to the strong overlap between locomotor categories and clades, fully disentangling these signals is not feasible, and we hence ran ordinary tests. Given that microanatomy is known to be plastic and highly dependent on an animal's lifestyle and shaped by functional demands (Buffrénil, Muizon, et al., 2021; Buffrénil, Ricqlès, et al., 2021; Kivell, 2016; Laurin et al., 2011), we interpreted our results as mostly reflecting functional constraints rather than phylogenetic heritage. We prioritized variables that are the least influenced by size and phylogeny, namely, global compactness, *P*, and *S*, and ran ordinary statistical tests for the rest of the analyses. Using the same timetree, we mapped global compactness across species (maximum likelihood; *contMap* function) based on their mean compactness value from dataset (A). We also mapped a single value of the global compactness acquired at the growth center for comparison.

Due to the large disparity of size in our sample (yellow‐bellied weasel, *Mustela kathiah*, 0.21 kg; Law et al., 2018, vs. southern elephant seal, *Mirounga leonina*, 1800 kg; Le Boeuf, 2021), we log‐transformed bone total length, minimal diaphyseal perimeter, and total diaphyseal volume.

Bone inner structure was investigated using two datasets. For each parameter, we tested whether our categories differed significantly, analyzing separately (A) the mean diaphyseal value of each species and (B) the variation along the bone shaft, using 100 values per species for Fiji parameters and 5 for BoneProfileR parameters. We controlled for normality and homogeneity of variance (shapiro.test and *leveneTest* functions) for each parameter before performing ANOVAs or ANCOVAs (Parameter ~ SizeProxy + Category; see below for the size proxy) on both datasets for each bone for the two levels of categories separately. ANOVAs were applied to dimensionless parameters (global compactness, resistance ratios *R*_CC_ and *R*_ML_, Parameters *P* and *S*); ANCOVAs were used for log‐transformed diaphyseal total volumes, with the size proxy as a covariate. For ANCOVAs, results are presented using boxplots of residuals from a linear regression with the size proxy. When significance was found (*p*‐value < 0.05), we conducted a pairwise post‐hoc analysis for the first‐level categories, using the Tukey–Kramer method to correct *p*‐values (*TukeyHSD* function). For dataset (A), the mean value of each category was tested against another category. For dataset (B), the same test was run on each of the 100 positions for Fiji's parameters and run on each of the five positions for BoneProfileR's parameters. To explore potential differences in bone structure among our categories, we first investigated variation in external shape using the bulkiness index and diaphyseal volume computed on the whole bone. Then, we tested if our categories were significantly different in their mechanical resistance using resistance ratios (SMA^1/4^ over CSA^1/2^) and in their bone distribution using global compactness, parameter *P*, and parameter *S*.

As our sample included species with a large size variation, we conducted a preliminary analysis to test whether groups differed significantly in their body size. We used a size proxy, calculated by taking the natural log of the sum of the minimum perimeters of the femur and humerus for each species following Campione and Evans (2012). We found that our categories (both sets of analyses) were significantly correlated (*p*‐value < 0.001) with our size proxy (Figure S2; Table S4). Generalist mustelids are significantly smaller than all the other categories. Semiaquatic mustelids are significantly smaller than all the other categories but larger than generalist mustelids. Since our sample splits into different size ranges, we tested Pearson's correlation (*cor.test* function) between each parameter and our size proxy. To confirm the validity of the association, we then plotted the data with fitted regression lines for each first‐level category. We did the same analysis with Pearson's correlation and fitted regression lines to compare the pattern of each parameter between femur and humerus.

## 3 RESULTS

### 3.1 External shape

#### 3.1.1 Bulkiness index (BI) and diaphyseal volume

**FIGURE 3:**
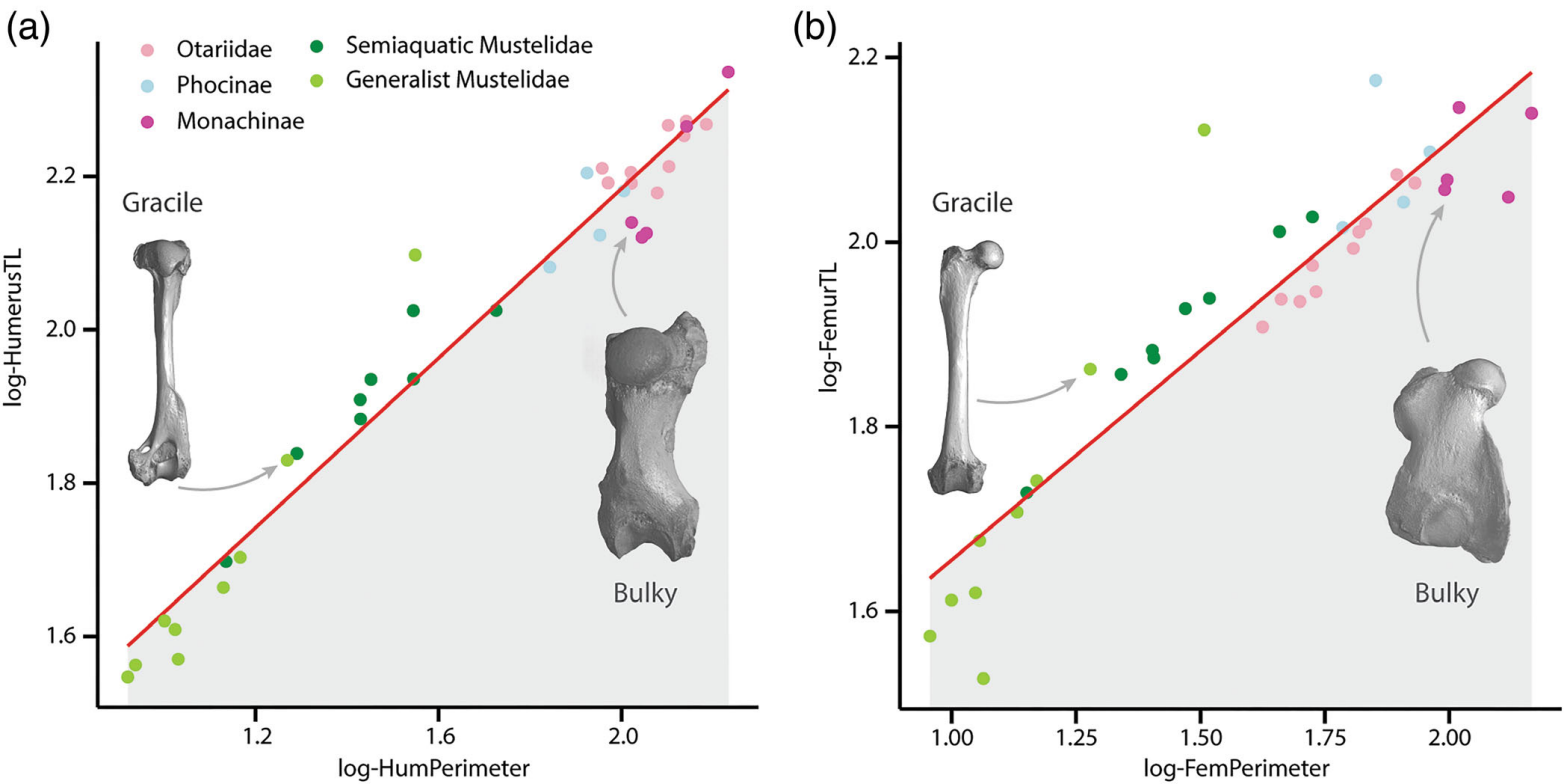
Bulkiness index: log‐transformed minimal diaphyseal perimeter against log‐transformed total length of the humerus (a) and femur (b) in pinnipeds and other selected carnivorans. The red line represents the fitted linear regression model across all taxa; above the line are the more gracile bones, below the lines are the bulkier bones. In (a) and (b), the bone on the left is from *Galictis vittata*, and the one on the right is from *Ommatophoca rossii* (not to scale).

The overall gracility of the humerus and femur varies greatly across the sampled carnivorans, especially that of the femur. Minimal bone perimeter against total bone length was plotted with a fitted linear regression line to represent the general scaling relationship across taxa. A bone with a wide perimeter and small total length is considered bulky, with a high bulkiness index (BI), while a longer bone with a smaller perimeter is described as gracile, that is, a lower BI (Figure 3; Table S3). Humeral values showed lower deviation from the linear model compared to those of the femur (Figure 3). For a similar humeral length, bone perimeter triples between a generalist mustelid and a monachine seal (Table S3; Figure 3a). Most semiaquatic mustelids plot above the regression line with low humeral BI (from 0.27 to 0.5, Table S3). The most gracile bones of the sample were found in generalist mustelids (BI from 0.24 to 0.29; Table S3). For a similar femoral length, most semiaquatic mustelids and some generalist mustelids fall well above the regression line, indicating relatively gracile bones (low BI_Femur_ circa 0.35, Table S3; Figure 3b) compared to the overall trend. Conversely, most otariids and phocids were found below the regression line, displaying bulkier bones with a perimeter representing around half the bone length (BI_Femur_ circa 0.55, Table S3). Monachine seals were mostly found below the regression line with the highest values of humeral BI (0.68–0.84, Table S3), indicating bulkier bones. Extremely bulky bones were found in the ross seal (*Ommatophoca rossii*) and the southern elephant seal (*Mirounga leonina*) that reached a femoral BI slightly above 1 (see Table S3 and Figure 3b), with a swollen, pachyostotic‐like aspect.

**FIGURE 4:**
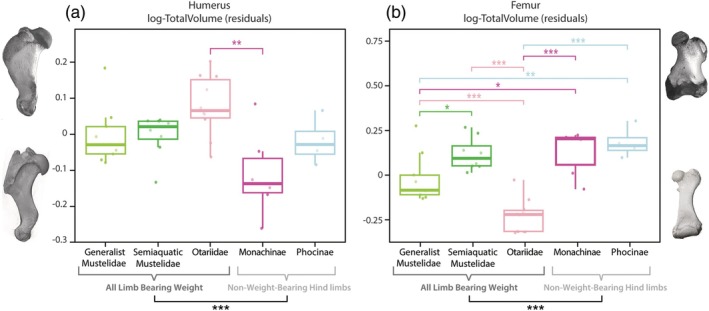
Log‐transformed and size‐corrected (residuals) diaphyseal total volume of humerus (a) and femur (b) per category. Colored bars correspond to pairwise comparisons among the first‐level categories (all groups separated), black bars for the second level of categories. *^,^**^,^***Significant differences with *p*‐value adj. < 0.05, 0.01, and 0.001 respectively (ANCOVA log(TotalVolume) ~ log(size proxy) + locomotor categories), ggsignif R package (Ahlmann‐Eltze & Patil, 2021). In (a), the bone at the top is from *Mirounga leonina* and the one at the bottom is from *Phoca vitulina*; in (b), the bone at the top is from *M. leonina*, and the one at the bottom is from *Arctocephalus gazella* (not to scale).

*Humerus*: In the humerus, the diaphyseal volume is highly influenced by the relative extent of the deltoid crest. Otariids, in particular, have a longer deltoid crest than the rest of the pinnipeds. Otariids showed the highest humeral volume for their size; it was found to be significantly higher than the humeral volume of monachines (Figure 4a). Comparisons between second‐level categories showed that phocids (with non‐weight‐bearing hind limbs) had significantly smaller humeral volume after size correction than the all‐limb weight‐bearing category (mustelids + otariids; Figure 4a,b).

*Femur*: Femoral volumes, corrected for size, were significantly lower in otariids compared to all four other groups (*p*‐value adj. < 0.001, Figure 4b). Generalist mustelids had significantly smaller femoral volumes than semiaquatic mustelids, monachines, and phocine seals (*p*‐value adj. < 0.037, <0.040, and <0.009 respectively). No difference was found between monachine and phocine seals. For the second‐level categories, non‐weight‐bearing hind limbs showed significantly larger femoral volumes than the all‐limb weight‐bearing category (after size correction *p*‐value adj. < 0.001, Figure 4b).

### 3.2 Internal anatomy

#### 3.2.1 Resistance to bending (SMA) versus compression (CSA)

##### Diaphyseal means

Resistance ratios, *R*_ML_ and *R*_CC_, of second moment of area to cross‐sectional area (SMA_ML_/CSA and SMA_CC_/CSA) were compared to assess whether the bones of certain groups reflect a predominance of resistance to bending over compression. A resistance ratio below 1.0 indicates a stronger resistance to axial compression (CSA) than to bending (along CC axis for SMA_ML_ or along ML axis for SMA_CC_, Kilbourne & Hutchinson, 2019).

**FIGURE 5:**
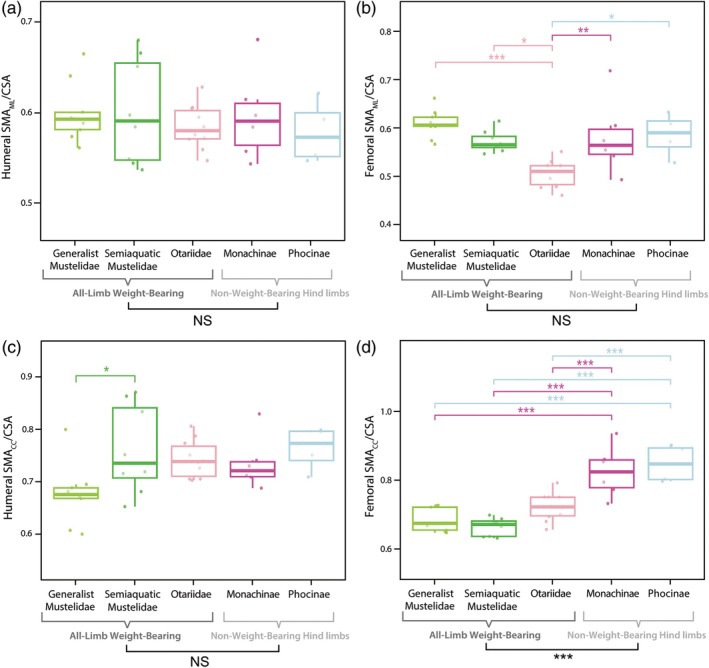
Resistance ratio of SMA^1/4^ to CSA^1/2^ (dimensionless) for the humerus (a, c) and the femur (b, d). Colored bars correspond to pairwise comparisons among the first level of categories (all classes separated), black bars correspond to the second‐level categories. NS: not significant, *p*‐value adj. > 0.05. *^,^**^,^***Significant differences with *p*‐value adj. < 0.05, 0.01, and 0.001 respectively (ANOVA: resistance ratio ~ locomotor categories).

Both *R*_ML_ and *R*_CC_ were found below 1.0, for humerus and femur, indicating a higher resistance to axial compression (CSA) than to bending (SMA) for all categories (Figure 5). Humeral and femoral *R*_CC_ were found to be higher than the respective *R*_ML_. For both ratios, the femur showed more inter‐category variation than the humerus.

*Humerus*: Humeri showed very limited variations among group means, including between the second‐level categories (Figure 5a,c). Only *R*_CC_ of generalist mustelids was found significantly lower than that of semiaquatic mustelids (*p*‐value adj. < 0.04; Table S4). The highest *R*_CC_ was found in the giant river otter (*Pteronura brasiliensis*), followed by the southern elephant seal (*Mirounga leonina*) and the oriental small‐clawed otter (*Aonyx cinerea*). The smallest *R*_CC_ was found in the marbled polecat (*Vormela peregusna*). For *R*_ML_, the elephant seal had the highest resistance ratio, well above the mean value of its category, followed closely by the small‐clawed otter. The smallest *R*_ML_ was found in the sea otter (*Enhydra lutris*) and the crabeater seal (*Lobodon carcinophaga*).

*Femur*: Femoral *R*_ML_ was found significantly lower in otariids compared to all the other categories (*p*‐value adj. < 0.001–0.05, Figure 5b,d). Otariids also had a lower *R*_CC_ than monachine and phocine seals (*p*‐value adj. < 0.004 and <0.001). Generalist and semiaquatic mustelids had lower *R*_CC_ than phocine and monachine seals (*p*‐value adj. < 0.001). For the second‐level categories, femoral *R*_ML_ showed no significant differences between the two groups. For *R*_CC_, the non‐weight‐bearing hind limb category had a significantly higher resistance ratio than the all‐limb weight‐bearing category. The highest *R*_ML_ was found in the southern elephant seal, clearly separated from the rest of the sample, while the smallest *R*_ML_ was in the Juan Fernández fur seal (*Arctocephalus philippii*). The highest *R*_CC_ was found in the elephant seal while the smallest *R*_CC_ was found in the marine otter (*Lontra felina*).

##### Diaphyseal profiles

Both resistance ratio R_CC_ and R_ML_ were found below 1.0 along the whole diaphysis of both humerus and femur.

**FIGURE 6:**
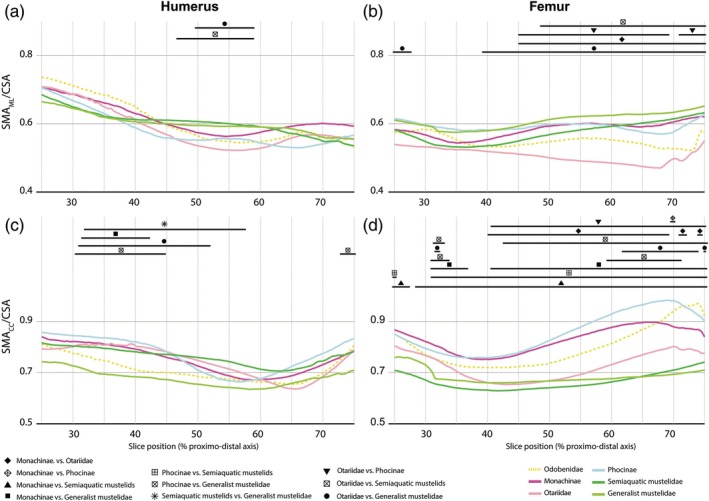
Proximo‐distal variation of resistance ratios, *R*_CC_ and *R*_ML_, in humerus (a, c) and femur (b, d). Resistance ratios were calculated with SMA^1/4^ over CSA^1/2^. Symbols indicate slice positions where a significant difference was found for a given pair‐wise comparison (adjusted *p*‐value < 0.05). Dotted yellow line represents the category consisting of only one species, the walrus, which was excluded from the statistical analyses. Each colored line represents the mean value of each locomotor category per slice.

*Humerus*: Humeral resistance ratios tend to decrease proceeding distally, with proximal and distal extremities of the diaphysis not showing significant distinctions among locomotor categories. For R_ML_, a limited number of positions in the middle section of the humerus (47%–58% of bone functional length (BL)) displayed significant differences (Figure 6a). Otariids have lower humeral R_ML_ compared to generalist and semiaquatic mustelids. Generalist mustelids have significantly lower humeral R_CC_ than all the other categories in the proximal first half of the bone (Figure 6c). The second‐level categories showed no significant differences in the humerus of all‐limb weight‐bearing against non‐weight‐bearing hind limb groups (Figure S3a,c).

*Femur*: For the femur, otariids have a significantly lower *R*_ML_ than all other categories, mostly in the distal half of the bone (Figure 6b). The femoral *R*_CC_ of otariids follows the same tendency as generalist and semiaquatic mustelids, with a significantly lower *R*_CC_ than the monachine and phocine seals (Figure 6d). Otariids were found to have a significantly higher femoral *R*_CC_ than generalist and semiaquatic mustelids, only in the distal portion of the diaphysis. Distally, otariids displayed an increasing femoral *R*_CC_, close to the level of monachines. A significant difference between all‐limb weight‐bearing versus non‐weight‐bearing hind limb was found only for *R*_CC_ (Figure S3b,d): the femoral *R*_CC_ is higher in non‐weight‐bearing hind limbs (*p*‐value adj. < 0.05 along the entire diaphysis).

*Comments on Odobenidae*: The walrus (*Odobenus rosmarus*) humerus has a *R*_ML_ similar to that of other pinnipeds, but a *R*_CC_ more comparable to generalist mustelids. Its femur features a *R*_ML_ intermediate between those of semiaquatic mustelids and otariids and a *R*_CC_ intermediate between those of otariids and phocids (Figures 6 and S3).

#### 3.2.2 Global compactness and medullocortical transition

##### Diaphyseal means: Global compactness (Cg), P, and S

No correlation was found between femoral global compactness and size (*p*‐value > 0.16). For the humerus, a significant correlation was recovered, but the regression violates several assumptions, and with a low *R* ^2^ (*p*‐value < 0.03, *R* ^2^ = 0.11; Figure S4a,b), we considered that there was no preponderant effect of size on global compactness. Parameter *P* had a small positive correlation with size in the humerus (*p*‐value < 0.01, *R* ^2^ = 0.22) and none in the femur (*p*‐value = 0.16). *P* followed the same pattern with size in femora and humeri as the mean compactness, also violating linearity (Figure S4c,d). For both mustelid categories, *P* remained relatively stable independently of size, while for all three pinniped categories, *P* scales positively with size (Figure S4c,d). Parameter *S* was found to positively correlate with size in the humerus and the femur (*p*‐value < 0.001, *R* ^2^ = 0.60, *p*‐value < 0.001, *R* ^2^ = 0.70 respectively). Parameter *S* increased with size for all categories except for phocine seals (Figure S4e,f). However, this regression was also overly influenced by outliers. A positive correlation between the humerus and the femur was found across our categories for global compactness and for *P* (*p*‐value < 0.05; Table S5), except in generalist mustelids, where the correlation was not significant (*p*‐value > 0.1; Table S5). For the four categories, Pearson's correlation coefficient was between 0.90 and 0.94 for global compactness and 0.79 and 0.94 for *P*. For parameter *S*, only generalist mustelids had a significant correlation of 0.81 (*p*‐value < 0.01; Table S5).

**FIGURE 7:**
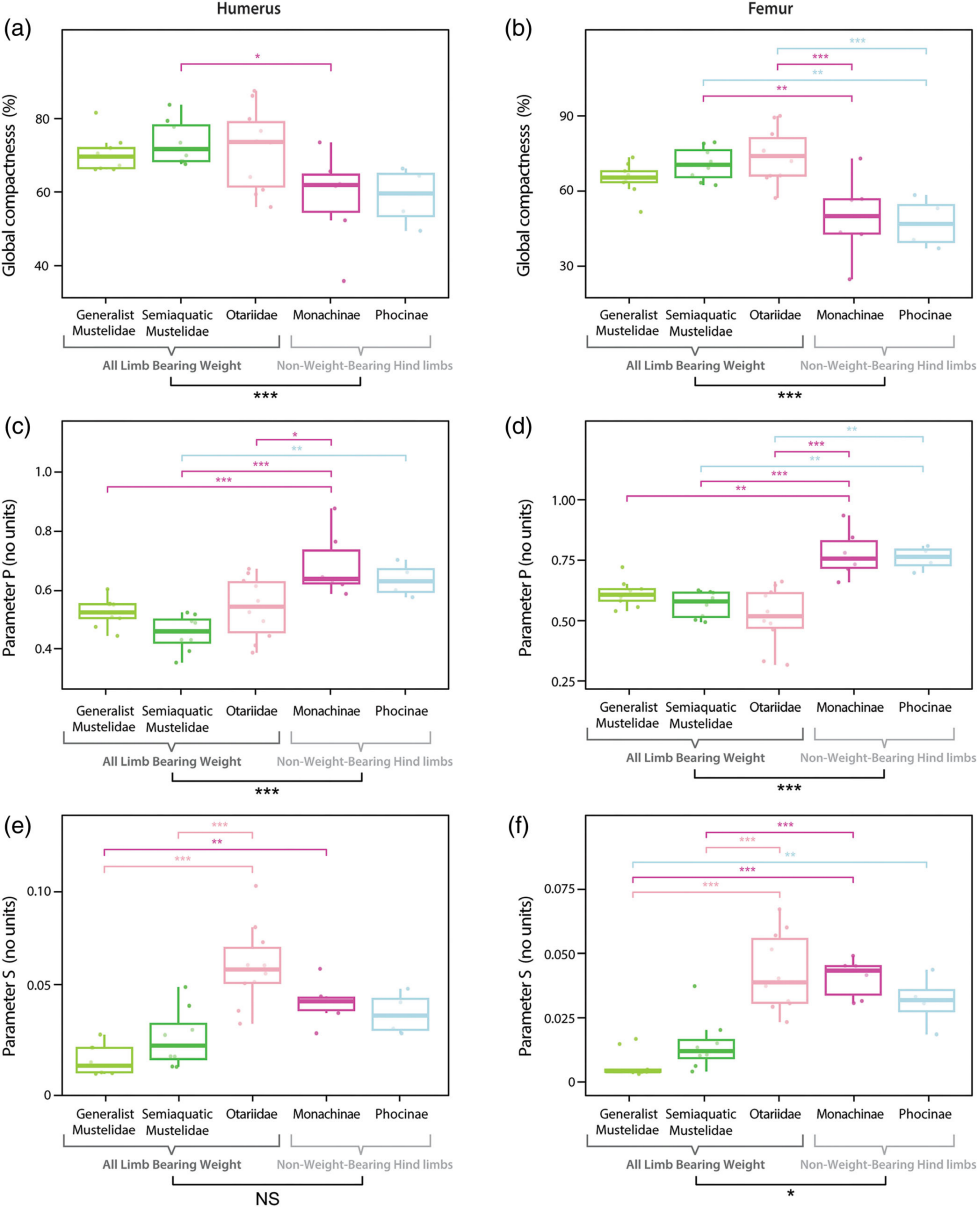
Global compactness, parameter *P* and *S* in humeri (a, c, e) and femora (b, d, f). Colored bars correspond to pairwise comparisons among the first‐level categories (all classes separated), black bars correspond to the second‐level categories. NS: not significant, *p*‐value adj. > 0.05. *^,^**^,^***Significant differences with *p*‐value adj < 0.05, 0.01, and 0.001 respectively (ANOVA: parameter ~ locomotor categories).

*Humerus*: The humerus of monachine seals displayed a significantly lower compactness and a higher *P* than in semiaquatic mustelids (*p*‐value adj. < 0.03 and <0.001; Table S4; Figure 7a,c). Monachine humerus was found with a higher *P* than that of otariids, semiaquatic, and generalist mustelids (*p*‐value adj. < 0.011, <0.001, <0.005, respectively). Phocine seals showed a higher humeral *P* than semiaquatic mustelids (*p*‐val adj < 0.008). Otariids had a significantly higher humeral *S* than semiaquatic and generalist mustelids (*p*‐val adj < 0.0001). Generalist mustelids also had a significantly lower *S* than monachine seals. No significant differences in global compactness, *P*, or *S* were found between phocine and monachine seals (Figure 7a,c,e). For the second‐level categories, phocids displayed a significantly lower humeral compactness with a higher *P* (*p*‐value adj < 0.0002 and <0.006) than the all‐limb weight‐bearing group. *S* was not found significantly different between the two groups (*p*‐value > 0.5). The least compact humerus was found in the southern elephant seal (Cg = 36%), associated with the highest *P* (87% the cross‐section occupied by the medullary region) and a high *S*, indicating a strong porosity. On the other hand, the most compact humerus was from the Juan Fernández fur seal (Cg = 88%), associated with very low *P* (0.38) and a high *S* (0.031).

*Femur*: For the femur, monachine and phocine seals had significantly lower mean compactness and a higher *P* than otariids and semiaquatic mustelids (*p*‐value adj. < 0.01–0.001, Table S4; Figure 7b,d). Monachines were also found with a significantly higher *P* than generalist mustelids (*p*‐value adj. < 0.007). Generalist mustelids were found with a significantly lower *S* than otariids, phocine, and monachine seals (*p*‐value adj. < 0.001–0.004). Semiaquatic mustelids were also found with a lower *S* than otariids and monachines (*p*‐value adj. < 0.001, Figure 7f). In the second‐level categories, phocids were found with significantly lower compactness, higher *P*, and higher *S* in their femur than the all‐limb weight‐bearing group (*p*‐value < 0.001–0.05). All phocids were found with a *P* above 0.65 (Figures 7d and 2). The elephant seal had the least compact femur (Cg = 25%) with the highest *P* (94% of medullary region occupancy) and the second highest *S*. The most compact femur was found in the subantarctic fur seal (*Arctocephalus tropicalis*, Cg = 90%) with a high *P* (0.33) and high *S* (0.067).

**FIGURE 8:**
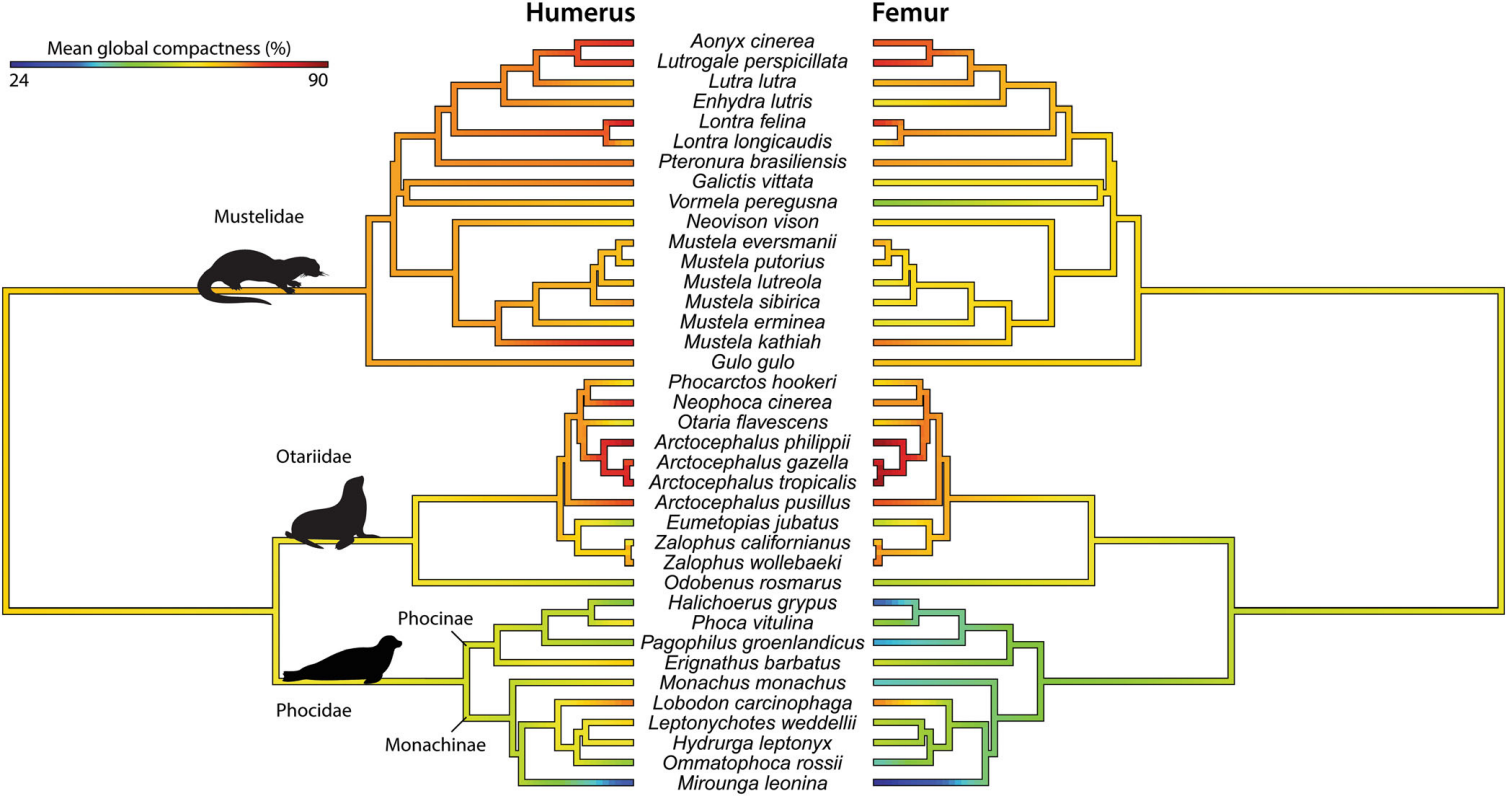
Phylogenetic mapping of the mean global compactness (%) acquired on the complete diaphysis of the humerus (left) and femur (right) for each species sampled (maximum likelihood estimation; *contMap* function, phytools package (Revell, 2012)).

**FIGURE 9:**
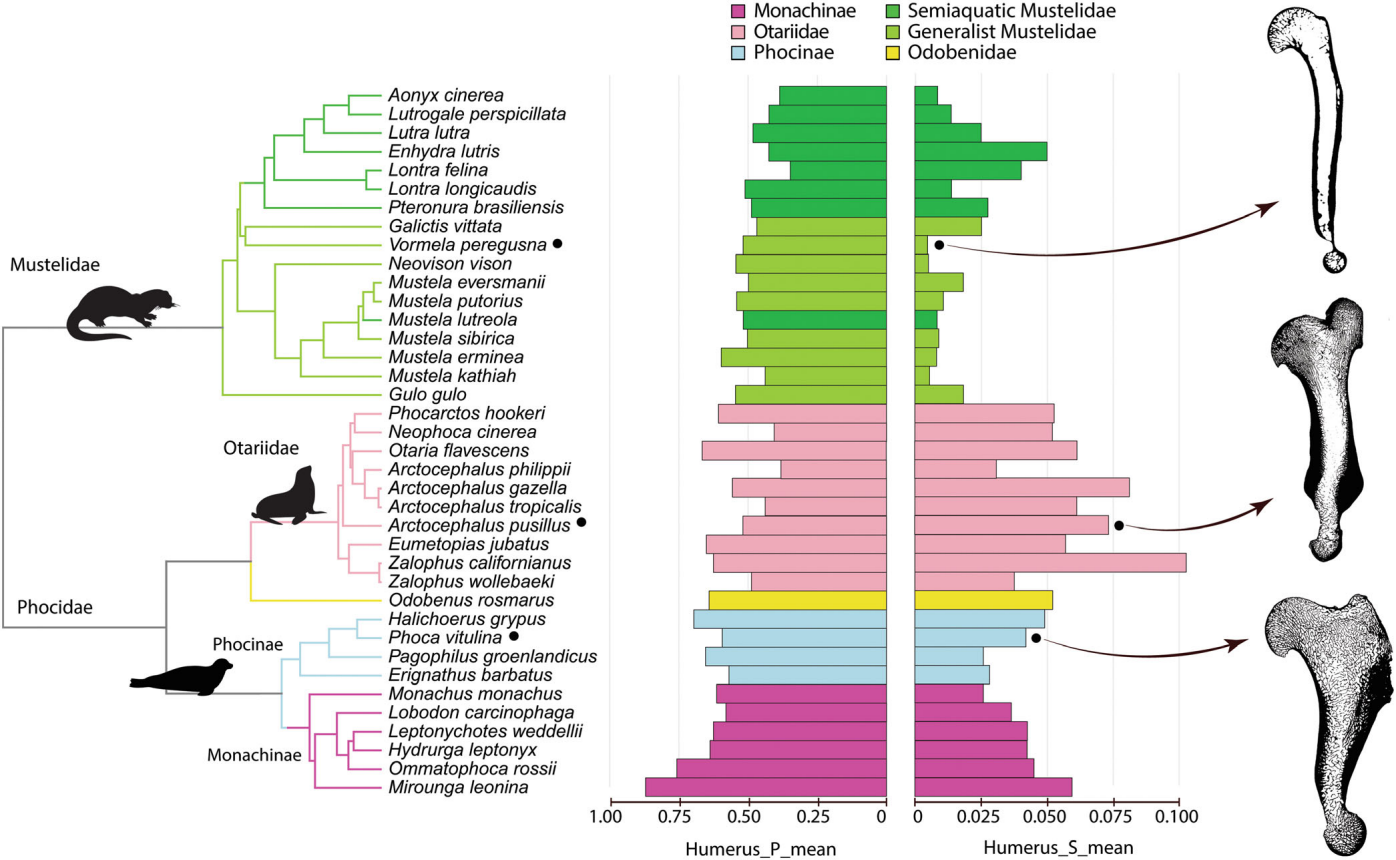
Bar plots for parameters *P* and *S* in all humeri sampled, colored by categories. Three species with similar values of *P* are shown in sagittal views (not to scale).

Compared to generalist mustelids, semiaquatic mustelids were found with more compact humeri and femora, a thicker cortex (low *P*), and a relatively abrupt medullocortical transition (low *S*) (Cg: 62%–84%, *P*: 0.35–0.63, S: 0.004–0.028). This pattern was more strongly expressed in some otariids that combine a thick cortex with a medullary region filled with trabeculae (high *S*) (Cg: 56.9%–90.1%, *P*: 0.32–0.66, *S*: 0.0042–0.050; Figures 8 and 9). Several phocine and monachine seals (*Halichoerus grypus, Pagophilus groenlandicus, Mirounga leonina*, and *Ommatophoca rossii*) displayed bones with very low compactness (25%–43%), a very thin cortex (high *P*), and a gradual medullocortical transition (high *S*). Between the two extremes, intermediate combinations were found with an association of medium to thick cortex with different levels of gradual medullocortical transition (Figures 9 and S5). Monachine and phocine seals, whose hind limbs are non‐weight‐bearing, display the most variations in terms of compactness in our sample; nevertheless, they have the least compact bones of the sample (Figures 7b and 8; Table 2).

**TABLE 2:** Summary of microanatomical parameters by clade.

Clade	Global compactness (Cg)	Parameter *P*	Parameter *S*
Mustelidae	62.4%–83.8%	0.35–0.62	0.004–0.028
Otariidae	56.0%–90.0%	0.32–0.66	0.029–0.10
Monachinae	24.8%–73.5%	0.58–0.94	0.026–0.059
Phocinae	37.1%–58.4%	0.57–0.81	0.018–0.049
Odobenidae	58.20%	0.68	0.046

When examined at the growth center, compactness values are clearly more homogeneously distributed, with overall higher values widespread across most taxa (Figure S6). Otariids differ from monachine and phocine seals in their femora, with otariids having the highest compactness, up to 90% (Figure S4a,b; see Figure 2 for sagittal cross‐sections of species with the lowest and highest femoral *P* in each locomotor category). Overall, the femur is less compact with a higher *P* than the humerus of the same species for all categories except in otariids that had the opposite pattern (Figures 8 and 9; Table S2). Parameter *S* was lower in the femur of otariids than in their respective humerus, and it did not show a specific pattern in the other categories. Parameter *S* was higher in pinnipeds than in mustelids (Figure 9). Extremely spongious bones were found in monachines, with a minimum femoral compactness found at 25% (Figure 8; Table S2).

##### Diaphyseal profiles: global compactness (Cg), P, and S

Humeral and femoral compactness profiles both reach a peak at their growth center, at around 60% of the humeral functional length and 37% of the femur. Several categories were significantly different from each other when areas away from the growth center were compared.

**FIGURE 10:**
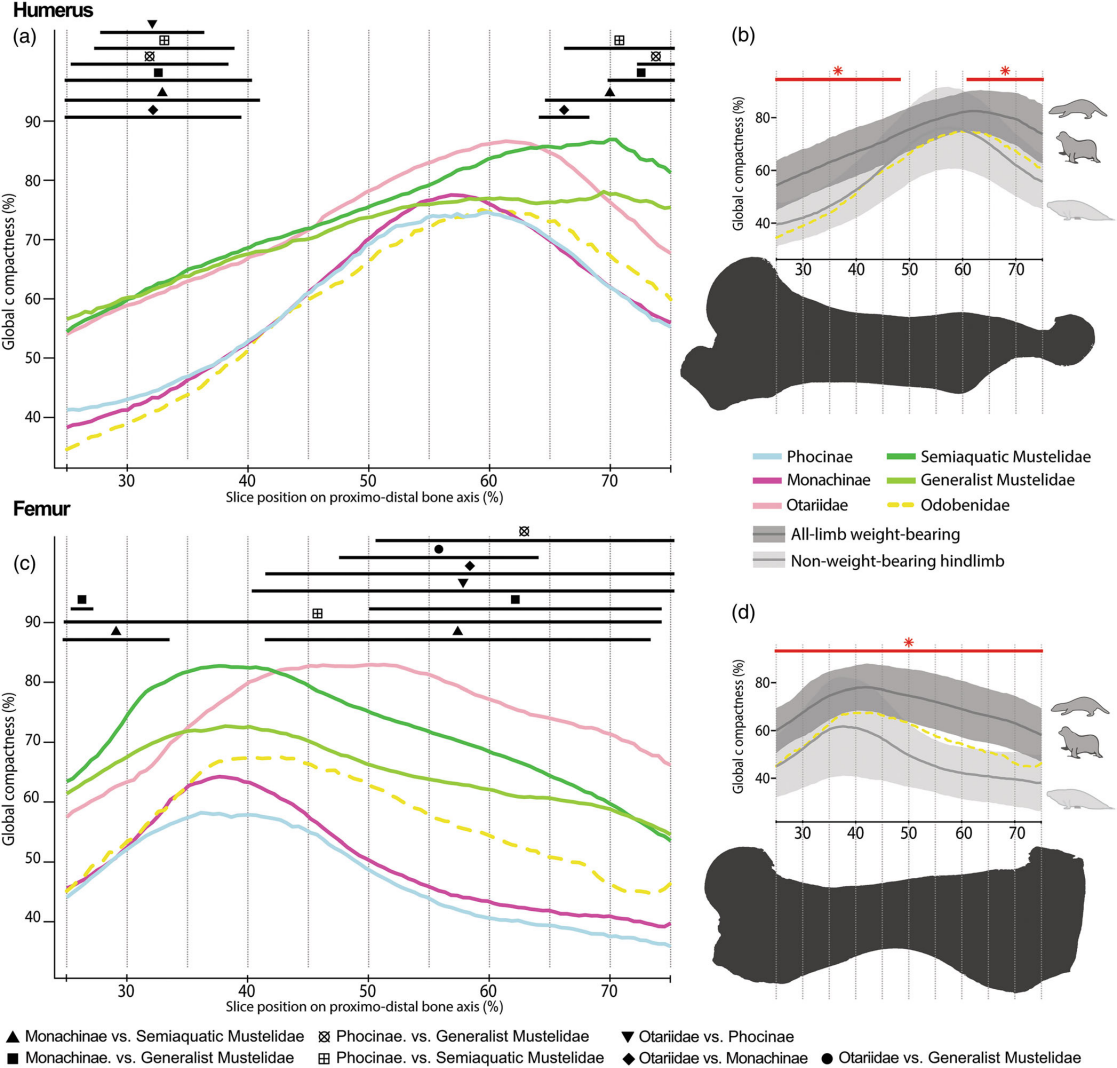
Compactness profiles (25%–75% of proximo‐distal functional length) in the humerus (a, b) and femur (c, d). Each line represents the mean value by category; symbols show positions where a significant difference (*p*‐value Tukey–Kramer adjusted) was found between two first‐level categories. Odobenidae was excluded from statistical tests. a–c and b–d correspond to the first‐ and second‐level categories, respectively. For the second‐level category comparison (b, d), thicker lines represent the mean and the shadow, the variance in each group; red line showing positions found significantly different.

**FIGURE 11:**
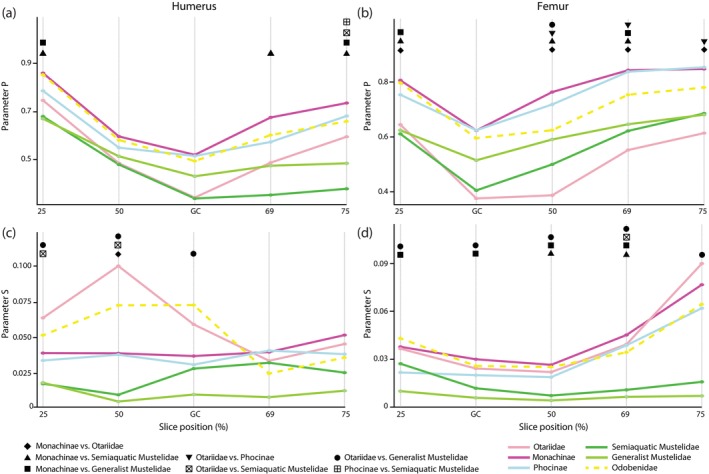
Bone medullocortical transition described by parameters *P* and *S* at five positions (percentage of bone functional length; GC, growth center) along the proximo‐distal axis of the humerus (a, c) and the femur (b, d). *P* represents the distance of the medullocortical transition from the center of the cross‐section (a high *P* indicates a wide medullary region, i.e., a thin cortex). *S* describes the transition; a low *S* indicates an abrupt transition, while a high *S* indicates a gradual one. Each colored line represents the mean value by category; symbols show positions where a significant difference (*p*‐value Tukey–Kramer adjusted) was found between two first‐level categories. Odobenidae, represented by one species, was excluded from statistical tests.

*Humerus*: The profile of humeral compactness sets apart mustelids and otariids from the other pinnipeds (Figure 10a). For phocine and monachine seals, compactness at the growth center is nearly twice as high as it is at 25% of the bone length. Distally to the growth center, compactness values decrease by ca. 26 percentage points (pp.) without reaching the lowest level of the proximal section. Compared to phocids, both mustelid categories and otariids exhibit a more gradual proximo‐distal increase in compactness, with generalists showing the weakest increase. Semiaquatic mustelids and otariids gain approximately 31 pp. of compactness in the bone's proximal half (Figure 10a). For these two groups, compactness decreases distally from the growth center, more markedly in otariids, while generalist mustelids show a plateau toward the distal end. For all categories, humeral profiles exhibit a strong increase in compactness, a strong decrease in *P*, and a relatively stable S from the proximal end to the growth center, except for otariids (Figures 10a and 11a,c). From the humerus' proximal end to its growth center (around 60% of BL), parameter *P* decreases from 0.74 to 0.34, showing a reduction in the size of the medullary region by almost half, that is, a cortex twice as thick.

The humeral proximal section (ranging from 25% to 40% of the bone length) was the most discriminant in the pair‐wise comparison. Proximally, phocine and monachine seals exhibit a significantly lower compactness and a lower *P* than generalist and semiaquatic mustelids. Distally to the growth center, these groups showed again significant differences in the same pattern as observed proximally (Figures 10a and 11a). Otariids show a significantly higher compactness and a lower *P* than phocine and monachine seals, proximally and distally to the growth center. Parameter *S* was only found to be significantly higher in otariids when compared to both mustelids and monachine seals in the humeral proximal section (*p*‐value adj. < 0.05 at 25% and 50% of BL and at the growth center, Figure 11c). Within mustelids, semiaquatic species do not have significantly different profiles from those of generalist mustelids. No differences were found between phocine and monachine seals (both with non‐weight‐bearing hind limbs). For the second‐level category, the all‐limb weight‐bearing category differs from phocids in having a humerus significantly more compact (*p*‐value adj. < 0.05 away from the growth; Figure 10b) and with a lower *P* (*p*‐value adj. < 0.05 at all five positions; Figure S7a). Parameter *S* was significantly higher in phocids only at 75% of BL (Figure S7c).

*Femur*: As for the humerus, femoral compactness profiles show two trends, one with an abrupt slope peaking at the growth center and the other peaking more gradually. The former represents phocids and the latter the all‐limb weight‐bearing category (Figure 10d). There is no clear separation between the femoral compactness profiles of pinnipeds and mustelids. The femur of monachine and phocine seals displays a strong compactness increase from 25% to around 35% of BL (i.e., the growth center). The increase was not as sharp as in the humerus. In the femur of phocine and monachine seals, compactness increases by 13–19 pp. relative to the proximal end. It decreases distally to the growth center, reaching, for all groups, lower levels than at the proximal end (Figure 10c). In otariids, semiaquatic and generalist mustelids, compactness increases by 23, 20, and 10 pp, respectively, from the proximal end to the growth center. For otariids and semiaquatic mustelids, it is associated with a decrease of *P* from circa 0.65 to circa 0.40, indicating a drastic reduction in the medullary region with a small decrease in *S*. In generalist mustelids, *S* was relatively stable throughout the whole diaphysis. For all first‐level categories, the distal half of the femur was the most discriminant region (distally to the growth center, at 50% and 69% of BL). All sampled species display a high peak of compactness close to the growth center, a position that was found not significant for distinguishing any of our first‐level categories, with one exception: at the growth center, phocine seals had significantly lower femoral compactness than semiaquatic mustelids. Otariids follow the pattern of semiaquatic mustelids; both have a higher compactness and lower *P* than phocine and monachine seals, both proximally and distally to the growth center (otariids *p*‐value < 0.05 on the 40%–75% region of BL, Figures 10c and 11b). Only distally to the growth center, otariids and semiaquatic mustelids were found to have a higher compactness and a lower *P* than generalist mustelids (*p*‐value < 0.05 48%–64% region of BL, Figure 10c and at 69% of BL in Figure 11b). Nonetheless, otariids and semiaquatic mustelids differ in parameter *S*. At 69% of BL, otariids have higher *S* values than semiaquatic mustelids (Figure 11d; see also Figure 2, sagittal views). Otariids' profile also has a more extended plateau of extreme compactness values than semiaquatic mustelids', distally to the growth center (Figure 10c). Generalist mustelid femora were found significantly more compact than those of phocine and monachine seals essentially from 50% to 75% of BL with a lower *P* than monachine seals only, at 25% and 69% of BL (Figures 10c and 11c). Generalist mustelids have a significantly lower *S* than monachine seals and otariids at all four femoral positions, except at 75% where it was only significantly lower than otariids (Figure 11d). Semiaquatic mustelids also have a significantly lower *S* than monachine seals at 50% and 69% of BL (Figure 11d). For the second‐level categories, phocids show significantly lower compactness levels, higher *P*, and higher *S* than the all‐limb weight‐bearing group along the whole femoral diaphysis (Figures 10d and S7b,d).

*Comments on Odobenidae*: The walrus' humerus follows the pattern of phocids, in terms of slope and in the range of compactness values and for parameter *P*. In its femur, however, it is intermediate, with a diaphyseal slope similar to all‐limb weight‐bearing species but with a level of compactness on the lower end of the group (Figure 10d), closer to phocids'. For both the humerus and the femur, parameter *S* is found in the range of that of otariids.

## 4 DISCUSSION

### 4.1 Disparity in bone microanatomy

Our results demonstrate that the microanatomy of the humerus and femur varies significantly across semiaquatic carnivorans. Our sample showed two trends in compactness and in the proportion of the medullary region, following the dichotomy of all‐limb weight‐bearing taxa (otariids and mustelids) and taxa with non‐weight‐bearing hind limbs (phocids). Furthermore, the humeral anatomy of the walrus proved to be similar to that of phocids, while the femoral one falls in between that of phocids and otariids. On one side of the spectrum, some otariids (i.e., the fur seals) and semiaquatic mustelids display the highest values of compactness and the smallest medullary region, with these features extending away from the growth center. This aligns with a pattern of bone mass increase (or increased compactness, known as osteosclerosis) compared to the terrestrial plesiomorphic condition, so far associated with shallow diving (less than 10 m deep) in mammals, like otters (Amson et al., 2014; Fish & Stein, 1991; Hays et al., 2007; Houssaye & Botton‐Divet, 2018). In our dataset, it was most clearly expressed in several otariids that combine a thicker cortex than mustelids with a medullary region extensively filled with trabeculae. This pattern was stronger in their femur than in their humerus. On the other end of the spectrum, some phocine and monachine seals exhibited very low compactness, with bone sections almost entirely occupied by the medullary region, filled by trabeculae—commonly described as an osteoporotic condition, characterized by skeletal lightening. This spongious, osteoporotic‐like pattern is strongly reminiscent of that found in the humerus of some modern cetaceans (Ricqlès & Buffrénil, 2001). Between these two trends, an intermediate group included species with an intermediate to thick cortex and a relatively gradual medullocortical transition, represented by several sea lions and some phocids.

The disparity among pinnipeds has never been quantitatively investigated in a systematic way. One notable exception is the work of Versaggi (1981), who sampled 10 pinniped species and the sea otter. His observations, though limited to individual thin sections and bone cuts, revealed distinct variation among pinnipeds—with similar patterns in both the humerus and femur—with some species exhibiting “increased structural density”, while others, like the elephant seal, showed highly cancellous bone “lacking discrete compact tissue”. Our study demonstrates the great disparity of microanatomical features both across pinnipeds and along long bones' diaphysis, which emphasizes that observations at a single level of the diaphysis might not capture the main differences in semiaquatic mammals. Indeed, the disparity of microanatomical patterns would not have been entirely captured by a single section or even a mean value of the complete bone. This is especially true if one had focused on the growth center (typically chosen as a standard sampling site in this type of study), as most sampled species tend to reach there a similarly compact structure (Figure 10). Historically, virtual or physical extraction of a slice at the growth center has been widely used for descriptive and analytical reasons (Amson et al., 2014; Canoville & Laurin, 2009; Gônet et al., 2023; Houssaye et al., 2015; Laurin et al., 2011; Meier et al., 2013; Simons et al., 2011). The growth center is the part of the bone that accumulates the most information in terms of growth layers (Francillon‐Vieillot et al., 1990); it displays essential information for histological investigations on species' life history. Nonetheless, this inherently highly compact section appeared less relevant in our study, which aimed to discriminate semiaquatic categories. The growth center strongly influenced diaphyseal profiles, and its position did not align with the midshaft; instead, its position varied slightly across species, as previously noted in other studies (Nakajima et al., 2014; Scheyer & Cerda, 2021). It was always distal to midshaft in the humerus (between 54% and 62% of BL) and proximal to midshaft in the femur (between 35% and 43% of BL). As a result, the cortex has a proximodistal hourglass‐shaped distribution in all species, except in those with highly spongious bones, where this pattern is not clearly visible. This distribution, where the growth center represents the most compact slice, can lead to an overestimation of bone compactness when its position is used to represent the entire shaft. A mean value for the whole bone proved to be a better representative, while diaphyseal profiles brought additional information. In the case of (semi)aquatic mammals, we would recommend avoiding a single slice analysis at the growth center for making locomotor inferences. Additionally, because bones with relatively low compactness are found in both terrestrial and fully aquatic species, parameters *P* and *S* proved essential to differentiate structural differences between the two groups (specifically regarding the proportion of the medullary region and presence or absence of porosity/trabeculae).

Across most groups, the humerus and femur exhibit similar microanatomical patterns, typically with the humerus being slightly more compact than the femur. Otariids, however, deviate from this trend, showing greater compactness in the femur (Table S3). This overall relationship is reflected in the strong correlation between the two bones (Table S5), regardless of whether the limb is primarily used for propulsion in the water. The shared pattern in stylopodia suggests that sampled species might be more constrained by their terrestrial locomotion, particularly because species with non‐weight‐bearing hind limbs have a limited use of their forelimbs in their terrestrial gait. For phocine seals, they are described as “friction hooks” (Kuhn & Frey, 2012) when most of their movement is done by the axial skeleton (Adam, 2005). In monachine seals, the forelimb can assist the caterpillar‐like movement on land or remain uninvolved when the seal uses a mediolateral undulation (O'Gorman, 1963). For all‐limb weight‐bearing species, despite a clumsy terrestrial gait for some otters and a restricted mobility for the hind limb of otariids, they all involve their fore‐ and hind limbs to bear their weight on land (Ray, 1963; Zellmer et al., 2021). The very strong correlation between humeral and femoral values of compactness and parameter *P* may also indicate that adaptations to a specific lifestyle influence bone microstructure systemically throughout the skeleton. Such a conclusion has already been reached in similar studies (Amson et al., 2018; Lieberman, 1996; Montañez‐Rivera & Hampe, 2020).

### 4.2 Light bone structure in phocids

All studied weight‐bearing bones (humerus and femur for mustelids and otariids, and humerus only in phocids) have a mean compactness roughly around 70% and *P* around 0.5 (with some outliers). On the other hand, the femur of phocids (non‐weight‐bearing) has a much less robust construction (compactness around 48%, *P* around 0.8 and a wide medullary region filled by trabeculae) with a lower resistance to axial compression than the humerus. Since the femur is integrated into the body contour of phocids, with only the distal limb visible externally as part of the hind flipper (Ray, 1963), its lighter bone structure compared to the humerus might reflect their differing roles on land and their differences in weight‐bearing constraints. Unlike the femur, the humerus can be involved in the terrestrial gait of phocids (O'Gorman, 1963). Despite the convergent external forelimb morphology between otariids and monachine seals (Hocking et al., 2021), our findings show that, on average, the humeral microanatomy of monachines does not differ from that of phocine seals. Nonetheless, certain monachines–such as the leopard seal (*Hydrurga leptonyx*) and, most notably, the crabeater seal (*Lobodon carcinophaga*)—fell into our intermediate group characterized by more compact bones and a thicker cortex than most monachines, reminiscent of some otariids (Figure 2). Furthermore, the walrus' humerus falls closer to the mean phocid profile while its femur has an intermediate position between the phocids and all‐limb weight‐bearing taxa (Figure 10). This pattern aligns with the walrus's lifestyle described as intermediate, as it is able to swim using both phocids' and otariids' styles, while having an otariid‐like locomotion on land (Gordon, 1981; Pierce et al., 2011).

Phocid seals have broader diving capacities (deeper diving depths and longer dive durations on average) than otariids (Jeanniard‐du‐Dot & Guinet, 2021). Previously, low bone density in the limb of cetaceans and the elephant seal was associated with deep diving (Stein, 1989; Wall, 1983). However, our findings showed that this feature is more widespread among pinnipeds than previously known, also occurring in species that dive to moderate depths, such as the harp seal (*Pagophilus groenlandicus*) and the gray seal (*Halichoerus grypus*) (Lydersen & Kovacs, 1993; Nordøy et al., 2008; Schreer et al., 2001; Sjöberg & Ball, 2000). When diving beyond several tens of meters, phocids' lungs, like cetaceans', can collapse, making their body denser than the surrounding water (Blix, 2018; Wall, 1983). In this situation, additional bone mass can be expected to be detrimental. Lung compression, including at moderate depths, allows the animal to dynamically regulate its buoyancy, providing greater adaptability when diving compared to species relying on dense bones for sinking. Our findings suggest that the presence of a light/spongious bone structure in pinnipeds cannot be only explained by deep diving. The extensive spongiosa, particularly evident in phocids, was already discussed for the deep‐diving southern elephant seal (*Mirounga leonina*) in Laurin et al. (2011), which recognized its structure to be reminiscent of that of extant cetaceans' humerus. Here, we saw that this pattern does not characterize all phocids; in fact, the elephant seal is, in our sample, the species where these features are most strongly expressed. It is closely followed by the Ross seal (*Ommatophoca rossii*), the gray seal (*Halichoerus grypus*), and the harp seal (*Pagophilus groenlandicus*). The three latter phocids feature the same pattern, but to a lesser extent: with slightly more compact bones and a cortex relatively thicker while having a similarly extended spongiosa with no open medullary region. The elephant seal, the Ross seal, and the Weddell seal (*Leptonychotes weddellii*) were found with the largest relative diaphyseal volume and the highest bulkiness index. They exhibited short and large bones with a swollen aspect, particularly in the femur. These species are capable of relatively deep dives but to different extents: the elephant seal can dive to almost 2400 m, with an average diving depth around 350–400 m (Costa et al., 2010; Le Boeuf, 2021; Muelbert et al., 2013; Schreer et al., 2001), whereas the Weddell seal's maximal dive was recorded at 600 m (Schusterman, 1981) with an average diving depth around 200 m (Schreer et al., 2001), and the Ross seal typically dives between 100 and 300 m (Blix & Nordøy, 2007). Despite this common behavior and a shared bulky aspect of their bones, the Weddell seal distinguishes itself from the other two seals by having more compact bones (included in our intermediate group) and spending less time at sea (between 55% and 85%, depending on the season; Boehme et al., 2016). The swollen aspect may be related to the extremely spongious internal structure and the need to withstand strong mechanical demands, bulkier gross morphology potentially compensating for the fragile structure of the bone. In pinnipeds, high bulkiness has only been described in association with osteosclerosis, which provides a clearer advantage for buoyancy control (Dewaele et al., 2019). However, no specific measurements or extensive comparative dataset exist to quantify pachyostosis. As pachyostosis alone was rarely discussed (Buffrénil et al., 2010), this type of bone growth in aquatic taxa requires further investigations.

Another constraint that appears to be associated with a lighter bone structure is the time spent at sea. Beyond a certain threshold—around 70%–80% of time at sea—an osteoporotic‐like phenotype was observed. In our sample, the southern elephant seal, the gray seal, the harp seal, the monk seal (*Monachus monachus*), and the Ross seal had the least compact femora, ranging from 24.8% to 43.6% in Cg values, with the elephant seal falling at the lower end of the range. These five species share a pattern of very extended time spent at sea. The Ross seal and the elephant seal return to shore only twice a year, once for breeding and once for molting, spending around 70%–80% of their time in the water (Arcalís‐Planas et al., 2015; Le Boeuf, 2021; Lewis & Eder, 2021; Loza et al., 2017). The elephant seal exhibits an exceptionally spongious bone, characterized by the absence of an open medullary region and an extremely thin cortex. Additionally, its femur exhibits the lowest resistance to axial compression relative to bending, suggesting a shift in loading constraints due to prolonged time in an environment without ground reaction forces, where axial compression becomes less relevant (Biewener, 1983; Gosnell et al., 2011). Although the inconsistency of documented data in the literature prevented a formal inclusion of time spent in water as an explanatory variable in our comparative analysis, the available data suggest a potential correlation between prolonged time spent at sea—rather than deep diving—and the loss of the typical terrestrial or semiaquatic bone structure pattern.

### 4.3 Bone mass increase in pinnipeds

A bone mass increase was identified in the femur of otariids, characterized by a relatively compact bone, a very thick cortex, and a more abrupt medullocortical transition than in the humerus, due to the femur's lower porosity. Relative to most phocids, otariids perform dives at moderate depths, around 15–130 m of maximum depth on average, depending on the species (Chilvers et al., 2006; Horning & Trillmich, 1997; Phillips, 2015; Schreer et al., 2001; Schusterman, 1981) with some species able to dive beyond 200 m (Renouf, 1991). The most aquatic of mustelids, the sea otter (*Enhydra lutris*), dives at shallower depths: at 30 m on average with a maximum recorded dive at 100 m of depth (Davis & Bodkin, 2021). Both the sea otter and otariids appear to rely on an osteosclerotic adaptation to facilitate their dives (Fish & Stein, 1991; Houssaye & Botton‐Divet, 2018; Nakajima & Endo, 2013; Tarasoff et al., 1972). Pinnipeds are even known to ingest stones, which might be another solution to actively regulate buoyancy (Wings, 2007). Interestingly, our findings reveal that the exacerbated pattern of bone mass increase in otariids can also be associated with deeper dives (ca. up to 100 m of depth) and a strong dependency on the terrestrial environment with a daily return to shore. Among our intermediate group, the crabeater seal and the leopard seal exhibit relatively moderate diving depths compared to the other phocids. The crabeater seal dives around 61 m deep on average (Bengtson & Stewart, 2018; Costa et al., 2010; Nordøy et al., 1995), while 90% of the leopard seal's dives are above 30 m (Krause et al., 2016). The latter is also known to have a shallower and briefer diving repertoire compared to other phocids (Drabek, 1975; Williams & Bryden, 1993). Notably, both species display higher bone compactness and a smaller medullary region than other true seals.

Two fur seals (*Arctocephalus philippii* and *A. tropicalis*) exhibited the highest compactness. Their medullary region was highly restricted, representing less than 34% of the bone section on average along the bone shaft. Otariids swim faster than other mammals of similar size, reaching speeds of around 1–2 m/s (Ladds et al., 2017; Ponganis et al., 1990; Watanabe et al., 2011). They are extremely agile hunters of fish and cephalopods (Llamazares‐Martín & Palagi, 2021). The fact that fast‐swimming animals that dive at moderate depths can have highly compact limb bones provides new perspectives on both previous and future interpretations of extinct species. This is particularly relevant to the evolution of early whales, as new (semi)aquatic species continue to be discovered and their past locomotion is still being investigated (Bebej & Smith, 2018; Bianucci et al., 2023; Gingerich et al., 2017, 2019; Houssaye et al., 2015; Lambert et al., 2019; Vautrin et al., 2020). The method used in this study could be applied to fossil specimens to investigate when these traits first emerged in the evolutionary history of various clades.

Among aquatic birds, penguins represent the most aquatic clade, with some species spending up to 70% of their time at sea. King penguins are known for high‐speed diving at around 50–250 m of depth on average (Ainley & Wilson, 2023). With their dependency on the terrestrial environment, they share similarities with otariids in their amphibious strategy. Penguins' long bones are osteosclerotic (Canoville et al., 2024; Habib & Ruff, 2008; Ksepka et al., 2015), and they may also regulate buoyancy behaviorally by ingesting stones (Wings, 2007). However, they differ from otariids in having air sacs and relatively rigid lungs (Ponganis et al., 2015), which therefore do not collapse as extensively under hydrostatic pressure as the lungs of pinnipeds. This condition has been proposed to account for the apparent discrepancy between the presence of osteosclerosis and the deep‐diving capabilities of these agile taxa (Canoville et al., 2024; Ksepka et al., 2015). Habib and Ruff (2008) suggested that the robust, flattened humerus of these forelimb‐propelled divers reflects the high mechanical loads experienced during aquatic propulsion, as these species rely exclusively on their forelimbs for aquatic propulsion, enhancing their resistance to strong bending forces. However, given that otariids and penguins exhibit a strong increase in bone mass in their femur—despite it not being involved in their main aquatic propulsion—we suggest that the combination of terrestrial constraints and buoyancy control is most likely the primary driver of this specialized phenotype, potentially leading to systemic adaptations across the skeleton.

### 4.4 Study limitations

Since the sex of most individuals of museum collections for our sample was not identified, this study only partially accounted for potential sexual dimorphism in the microanatomy of the humerus and femur (Figure S1). In pinnipeds, males tend to be larger than females, with some behavioral differences (Berta, 2018). To mitigate potential bias, we used size‐independent or size‐corrected variables, and we assumed that intraspecific variation was lower than interspecific variation. Several specimens were sampled for most species, and in all cases, the individual values lie in the vicinity of the group's other individuals (Figure S1).

Given the intricate relationship between locomotor categories and phylogeny in pinnipeds, fully disentangling their respective influences on microanatomical variation was not possible. A phylogenetic signal might still have influenced some of our results, but our findings did correspond to known microanatomical characteristics found in other semiaquatic and fully aquatic tetrapods (Amson et al., 2014; Canoville et al., 2021; Houssaye & Botton‐Divet, 2018; Houssaye & Buffrénil, 2021; Houssaye & Fish, 2016; Montoya‐Sanhueza & Chinsamy, 2017; Nieminen et al., 2024). Our functional interpretations remain applicable at least to the taxa sampled in this study. A potential avenue to support our findings would be to incorporate cetaceans with diverse swimming behaviors (deep diving, pelagic, and surface feeders) into a comparative analysis. Given that the effects of deep diving on bone mass decrease remain debated (this study; Lambert et al., 2011; Rolvien et al., 2017; Zotti et al., 2009) and considering that elephant seals rival beaked whales in achieving the deepest recorded dives (Schorr et al., 2014; Tyack et al., 2006), such comparisons could offer valuable insights into microanatomical functional adaptations.

## 5 CONCLUSION

Our findings showed that the diversity found in humeral and femoral bone structure among pinnipeds and mustelids can be linked to functional aspects of the animals' lifestyles, most notably for the femur. Following our expectations, the non‐weight‐bearing hind limb category (phocids) displayed a lower resistance to axial compression in the femur than to bending along the mediolateral axis, compared to the all‐limb weight‐bearing category (otariids + mustelids). Additionally, in phocids, the femur exhibited lower resistance to axial compression than the humerus, likely reflecting its limited terrestrial function and lack of weight‐bearing ability. Contrary to our expectations, no structural pattern was found between the humerus and femur in relation to their role in the animal's aquatic propulsion. Only the femur of otariids exhibited a stronger resistance toward axial compression compared to their humerus. Contrary to our expectations, the femur and humerus exhibited correlated patterns in compactness and cortical thickness in each of the categories; therefore, our functional interpretation aligns with a potential systemic skeletal adaptation of the microstructure.

Our findings reveal a diversity of microanatomical patterns in pinnipeds much broader than previously appreciated, illustrating their varied adaptations as semiaquatic mammals. Extensive time spent at sea (or reduced time on land) appears to have driven the selection for an osteoporotic‐like bone pattern in species that are still reliant on the terrestrial environment. Finally, this investigation challenges the traditional association between bone mass increase and both shallow diving and slow swimming, by extending it to semiaquatic species that dive at moderate depths with great agility. Similar patterns have previously been observed in penguins—fast, agile swimmers like otariids—whose increased bone mass contrasts with that of non‐swimming relatives.

To conclude, our approach provides an improved framework for better characterizing semiaquatic locomotor categories, with potential applicability beyond Carnivora. It offers a methodological basis for making more refined inferences on the locomotion of extinct semiaquatic species.

## AUTHOR CONTRIBUTIONS

**Apolline Alfsen:** Writing – original draft; conceptualization; methodology; software; investigation; writing – review and editing; data curation; formal analysis; visualization; funding acquisition; resources; validation. **Christian de Muizon:** Writing – review and editing; funding acquisition. **Olivier Lambert:** Writing – review and editing; funding acquisition. **Giovanni Bianucci:** Writing – review and editing; funding acquisition. **Antonia R. Kaffler:** Resources; writing – review and editing. **Matthew R. McCurry:** Writing – review and editing; resources. **Alexandra Houssaye:** Resources; writing – review and editing. **Rafael M. Varas‐Malca:** Writing – review and editing. **Rodolfo Salas‐Gismondi:** Writing – review and editing. **Oliver Hampe:** Writing – review and editing; funding acquisition; supervision; resources; project administration. **Eli Amson:** Writing – review and editing; supervision; methodology; validation; project administration; funding acquisition; resources.

## CONFLICT OF INTEREST STATEMENT

The authors declare no conflicts of interest.

## Supporting information


**FIGURE S1:** Principal component analysis (PCA) on 12 structural parameters of the femur and humerus (six parameters for each bone: global compactness, Parameter *P*, Parameter *S*, *R*_ML_, *R*_CC_, and diaphyseal volume), using a mean value by species (a) or with each individual (b). For some individuals the sex was known (F, female; M, male; NA, unknown).

**FIGURE S2:** Size proxy (log). Colored bars correspond to pairwise comparisons among the first‐level categories (all classes separated), black bars correspond to the second‐level categories. ***Significant differences with *p*‐values Tukey–Kramer adjusted <0.001 (ANOVA: Sizeproxy ~ Categories). See Table S4 for post‐hoc summary results.

**FIGURE S3:** Proximo‐distal variation of resistance ratios (dimensionless), *R*_CC_ and *R*_ML_, in humerus (a, c) and femur (b, d). Resistance ratios were calculated with SMA^1/4^ over CSA^1/2^. Red stars indicate slice positions where a significant difference (adjusted *p*‐value < 0.05). Dotted yellow line represents the category consisting of only one species, the walrus, which was excluded from the statistical analyses. Thick lines represent the mean and the shadow, the variance in each group.

**FIGURE S4:** Linear regression models by categories (parameter ~ size proxy) in the humerus (a, c, e) and in the femur (b, d, f ).

**FIGURE S5:** Bar plots for parameters *P* and *S* in all femora sampled, colored by categories.

**FIGURE S6:** Phylogenetic mapping of the global compactness (%) acquired at the growth center of the humerus (left) and the femur (right) for each species sampled (maximum likelihood estimation; *contMap* function, *phytools* package (Revell, 2012)).

**FIGURE S7:** Bone medullocortical transition described by parameters *P* and *S* at five positions (percentage of bone functional length; GC, growth center) along the proximo‐distal axis of the humerus (a, c) and the femur (b, d). *P* represents the distance of the medullocortical transition from the center of the cross‐section (a high *P* indicates a wide medullary region, i.e., a thin cortex). *S* describes the transition; a low *S* indicates an abrupt transition while a high *S* indicates a gradual one. Thick lines represent the mean value by category, and the shadow represents the variance in each group; red stars show positions where a significant difference (*p*‐value T–K adjusted) was found between the second‐level categories.

**TABLE S1:** Institutional abbreviations (catalogue abbreviation when different).
**TABLE S2:** Specimen list with affiliations.
**TABLE S3:** Bulkiness index (BI) calculated by dividing bone minimal perimeter (MinPerim) by bone total length (TL); variation of global compactness (Cg) along the diaphysis with minimal, maximal and mean value (minCg, maxCg, meanCg, respectively). Sorted by category then by species.
**TABLE S4:** Summary tables of ANOVAs and ANCOVAs for all parameters and the size proxy.
**TABLE S5:** Pearson's correlation test between the femur and the humerus.
**TABLE S6:** ZIP archive containing all CSV files with Fiji‐acquired parameters for each bone of every specimen.
**TABLE S7:** CSV files of BoneProfileR‐acquired parameters for each five slices of each bone in every specimen.

## Data Availability

All raw data are available in the supplementary online material.
