# Supplementary figures and images for: Broadening the semiaquatic scene: Quantification of long bone microanatomy across pinnipeds
Source: Anat Rec (Hoboken). 2025 Oct 13;309(8):2124–50. doi: 10.1002/ar.70058 (PMC13331545; doi:10.1002/ar.70058)

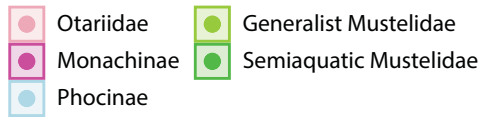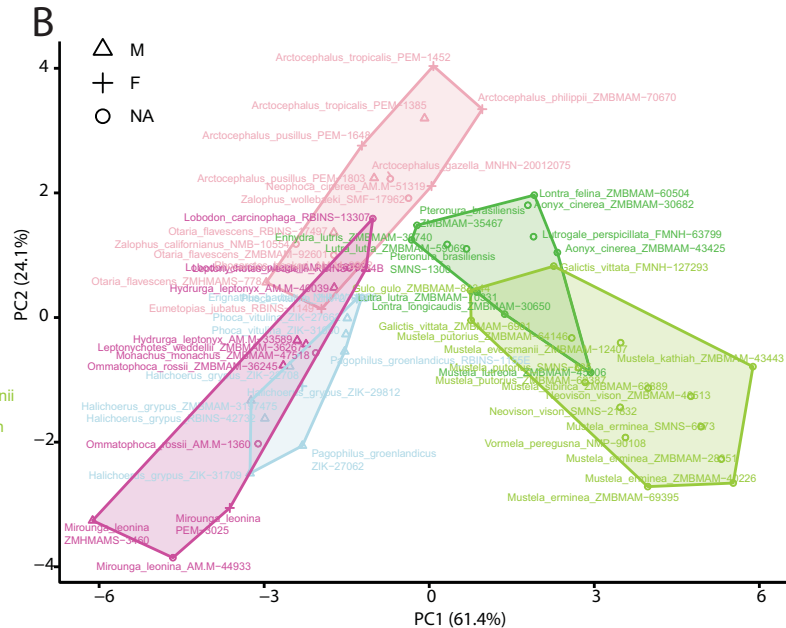

Supplement: Supplementary file 1 — FIGURE S1: Principal component analysis (PCA) on 12 structural parameters of the femur and humerus (six parameters for each bone: global compactness, Parameter P, Parameter S, R ML, R CC, and diaphyseal volume), using a mean value by species (a) or with each individual (b). For some individuals the sex was known (F, female; M, male; NA, unknown). [file AR-309-2124-s007.pdf]

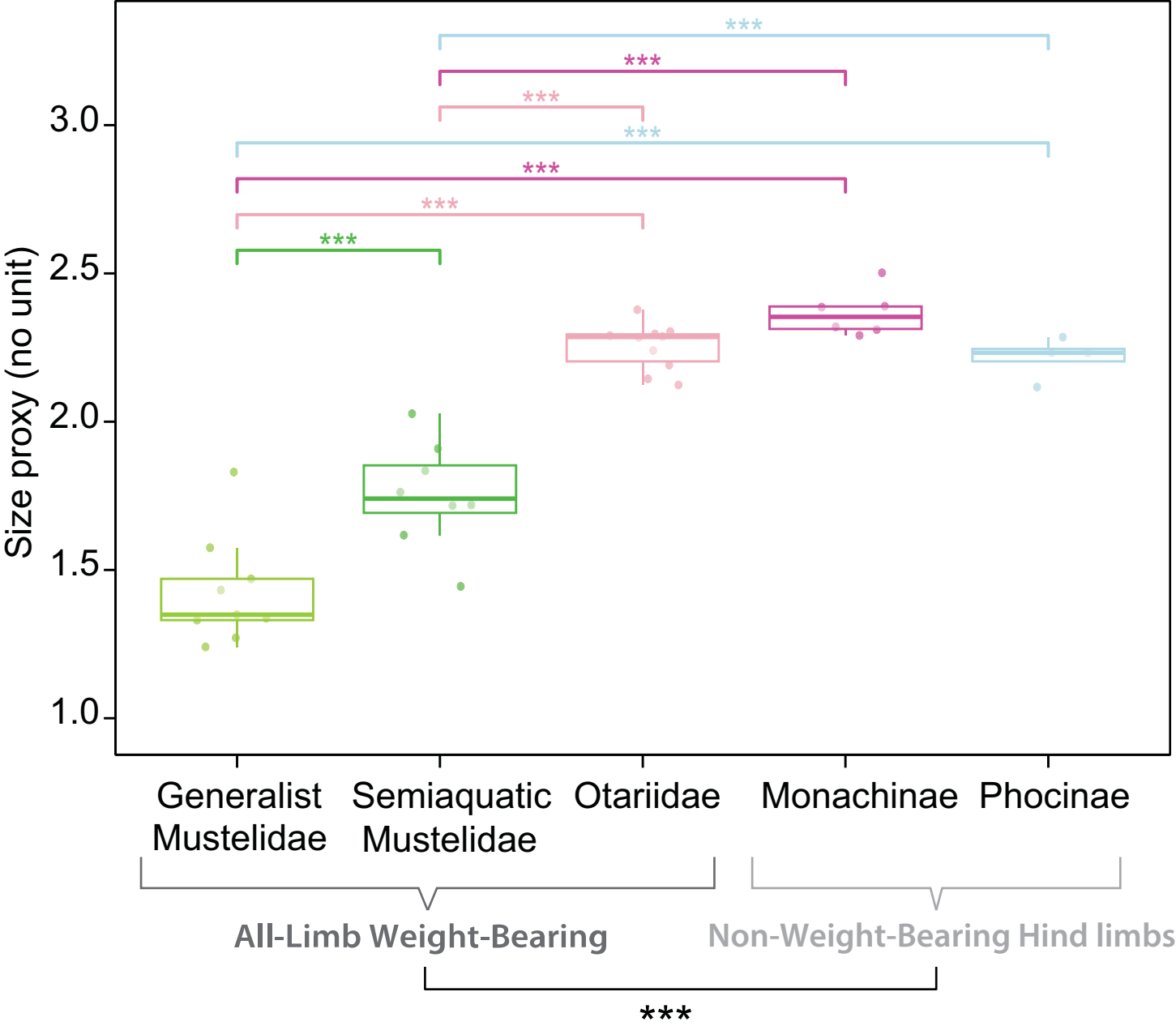

Supplement: Supplementary file 2 — FIGURE S2: Size proxy (log). Colored bars correspond to pairwise comparisons among the first‐level categories (all classes separated), black bars correspond to the second‐level categories. ***Significant differences with p‐values Tukey–Kramer adjusted <0.001 (ANOVA: Sizeproxy ~ Categories). See Table S4 for post‐hoc summary results. [file AR-309-2124-s001.pdf]

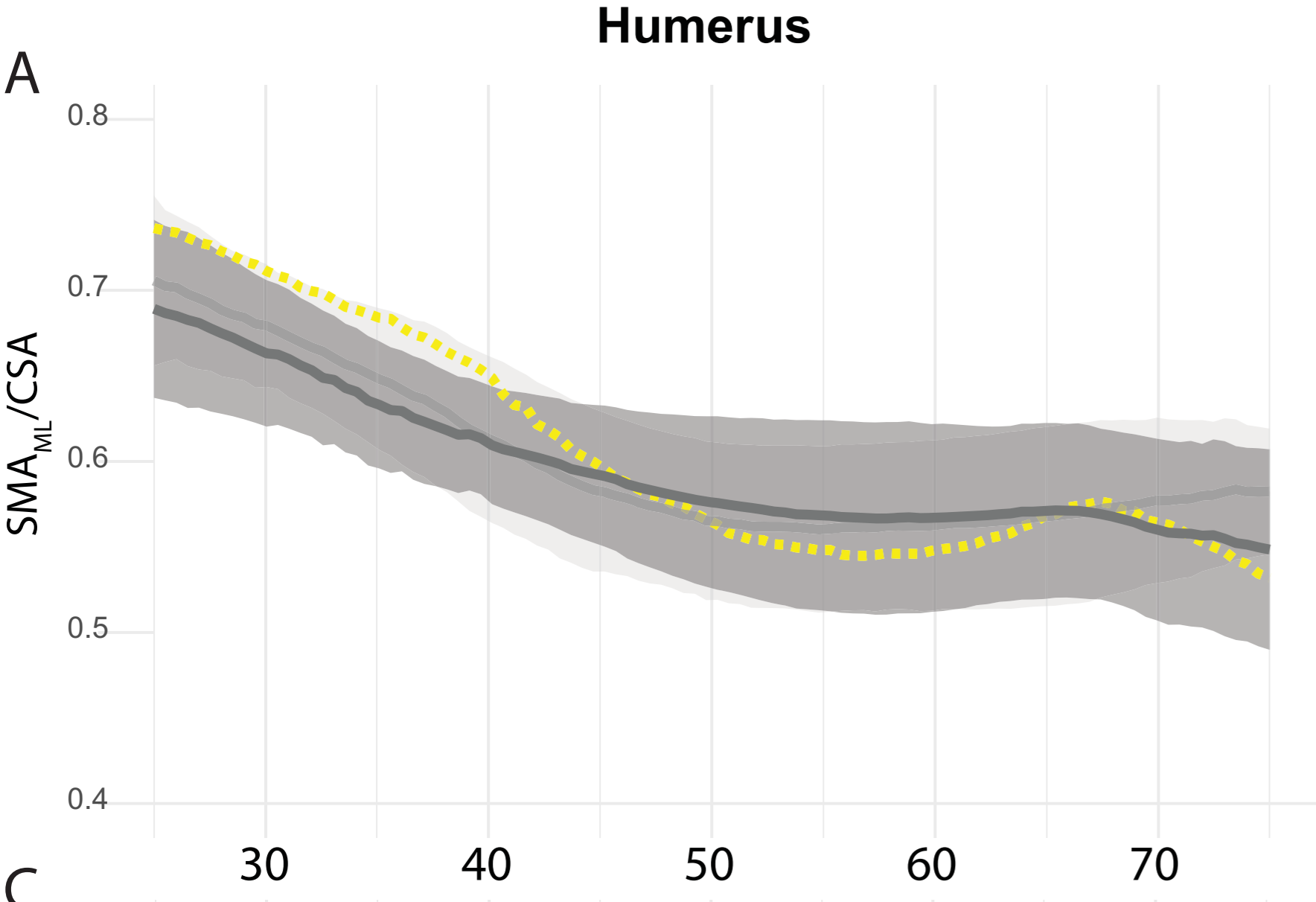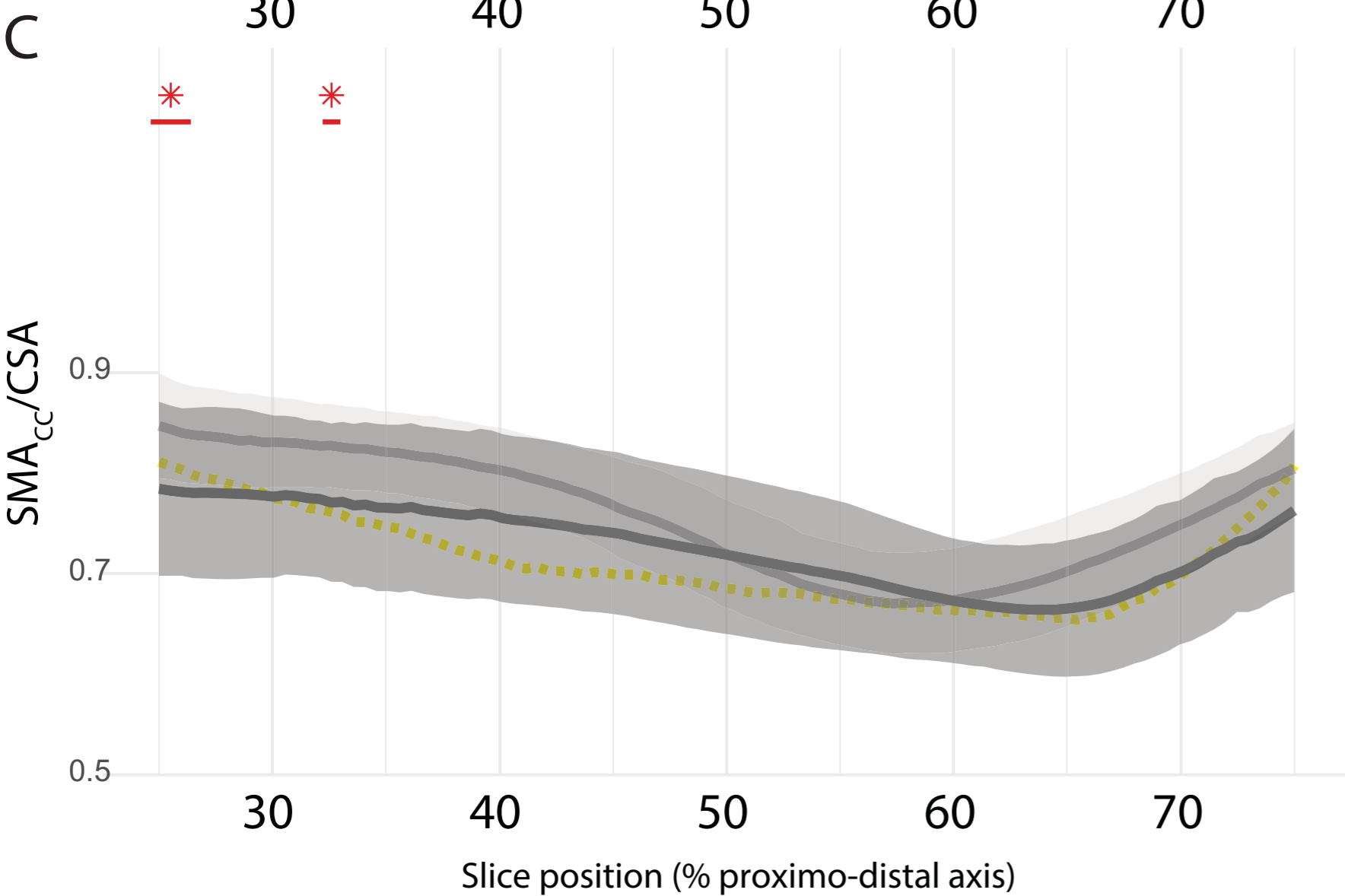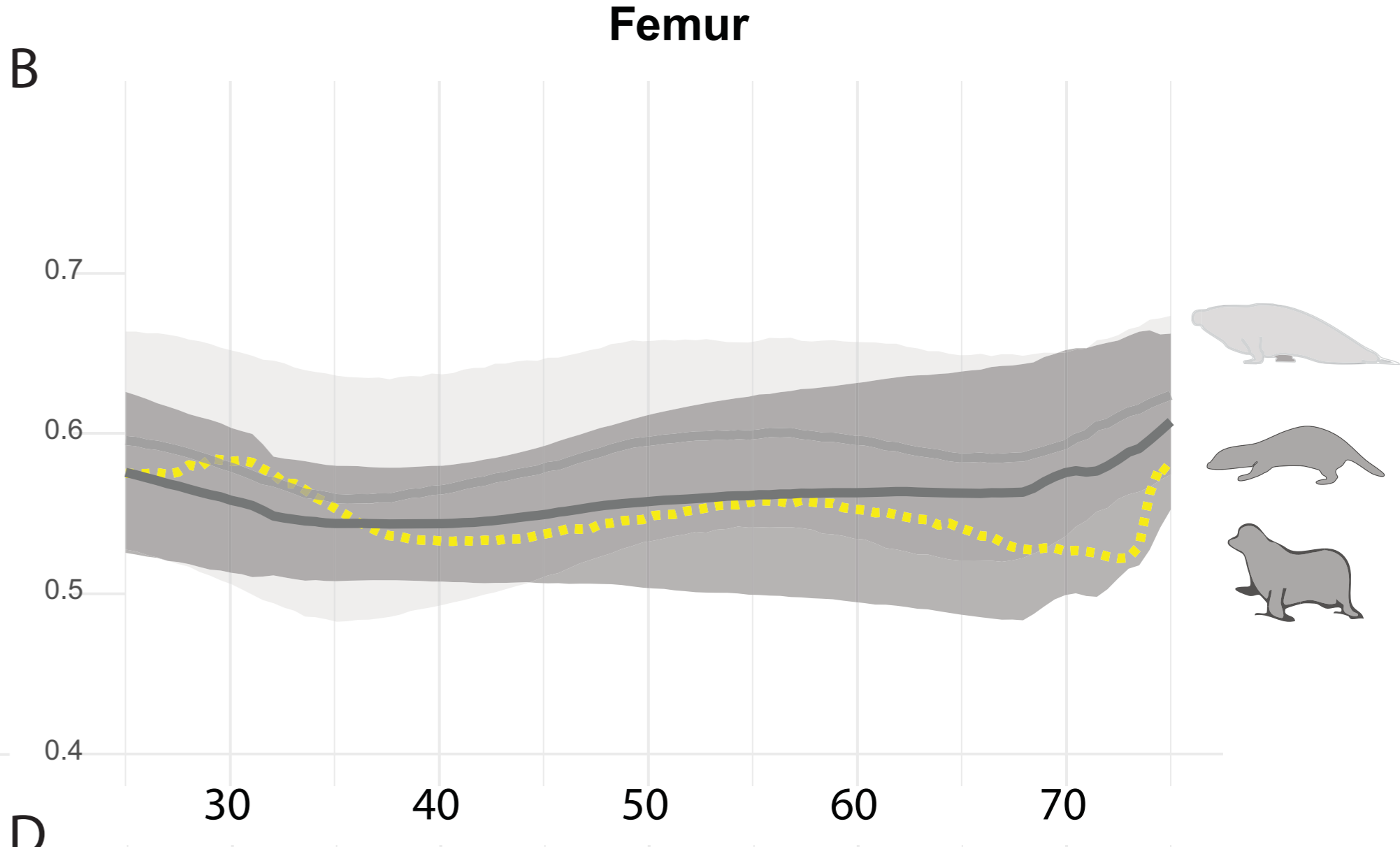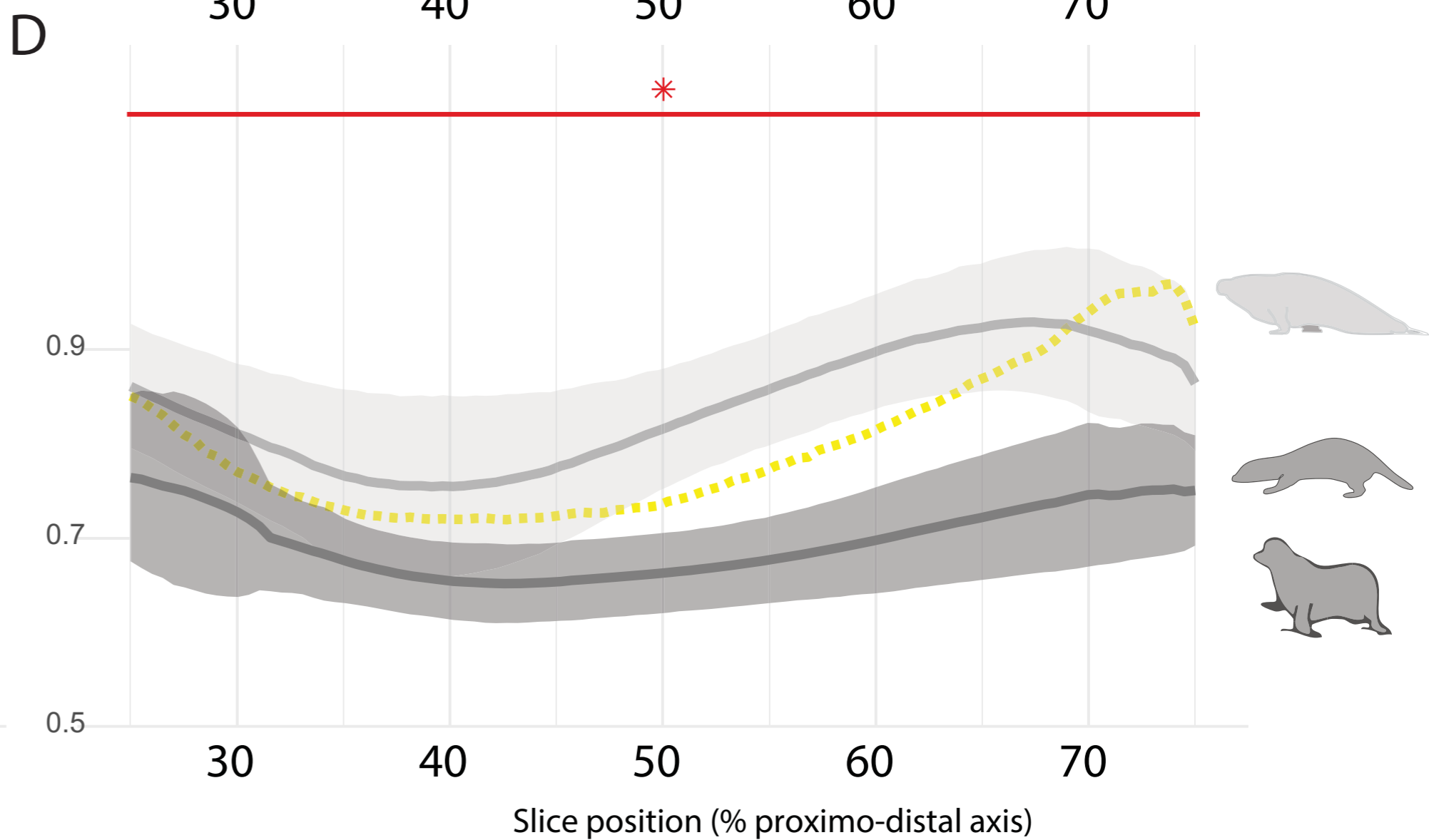

— All-limb weight-bearing — Odobenidae  
— Non-weight-bearing hindlimbs

Supplement: Supplementary file 3 — FIGURE S3: Proximo‐distal variation of resistance ratios (dimensionless), R CC and R ML, in humerus (a, c) and femur (b, d). Resistance ratios were calculated with SMA1/4 over CSA1/2. Red stars indicate slice positions where a significant difference (adjusted p‐value < 0.05). Dotted yellow line represents the category consisting of only one species, the walrus, which was excluded from the statistical analyses. Thick lines represent the mean and the shadow, the variance in each group. [file AR-309-2124-s008.pdf]

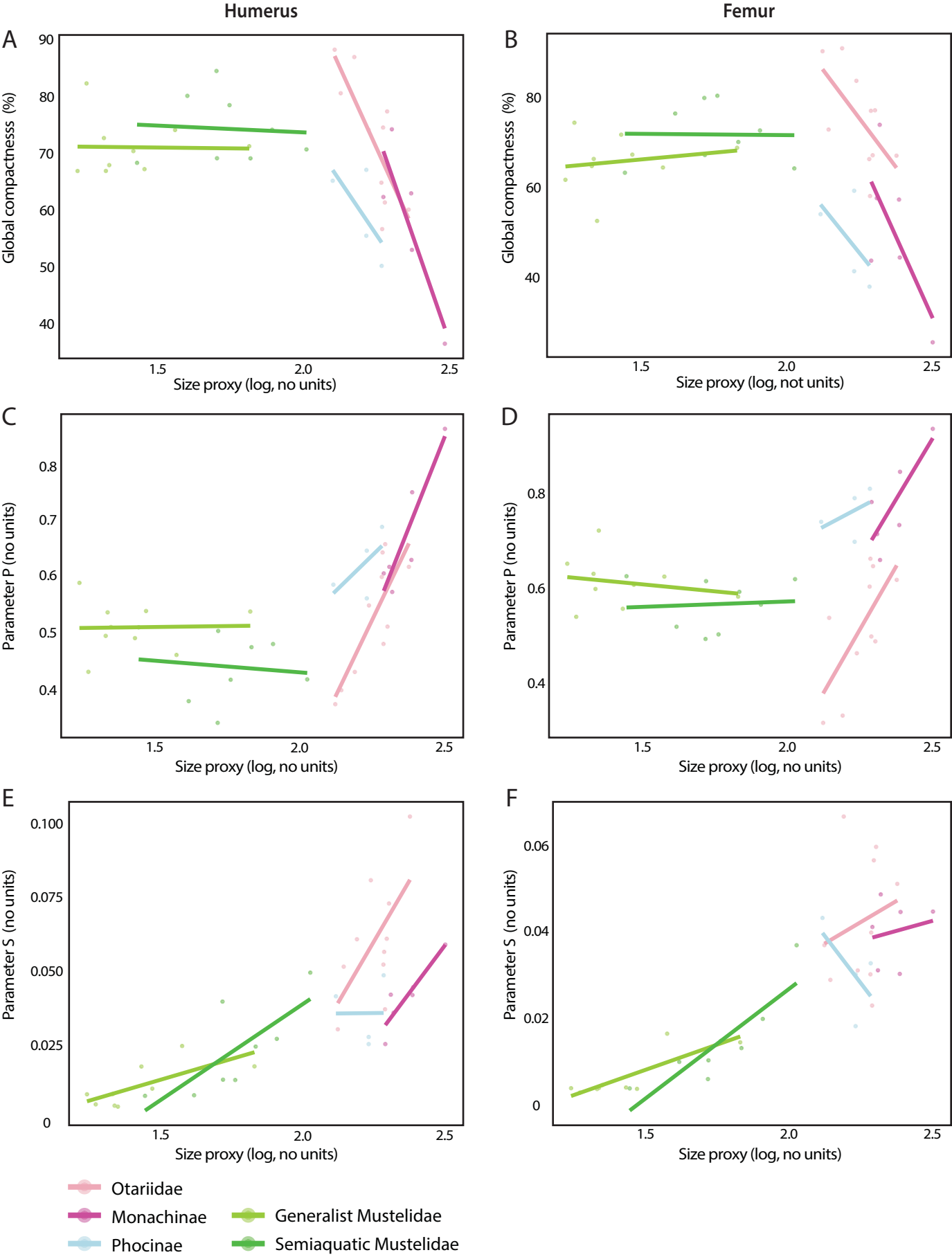

Supplement: Supplementary file 4 — FIGURE S4: Linear regression models by categories (parameter ~ size proxy) in the humerus (a, c, e) and in the femur (b, d, f ). [file AR-309-2124-s005.pdf]

# Femur

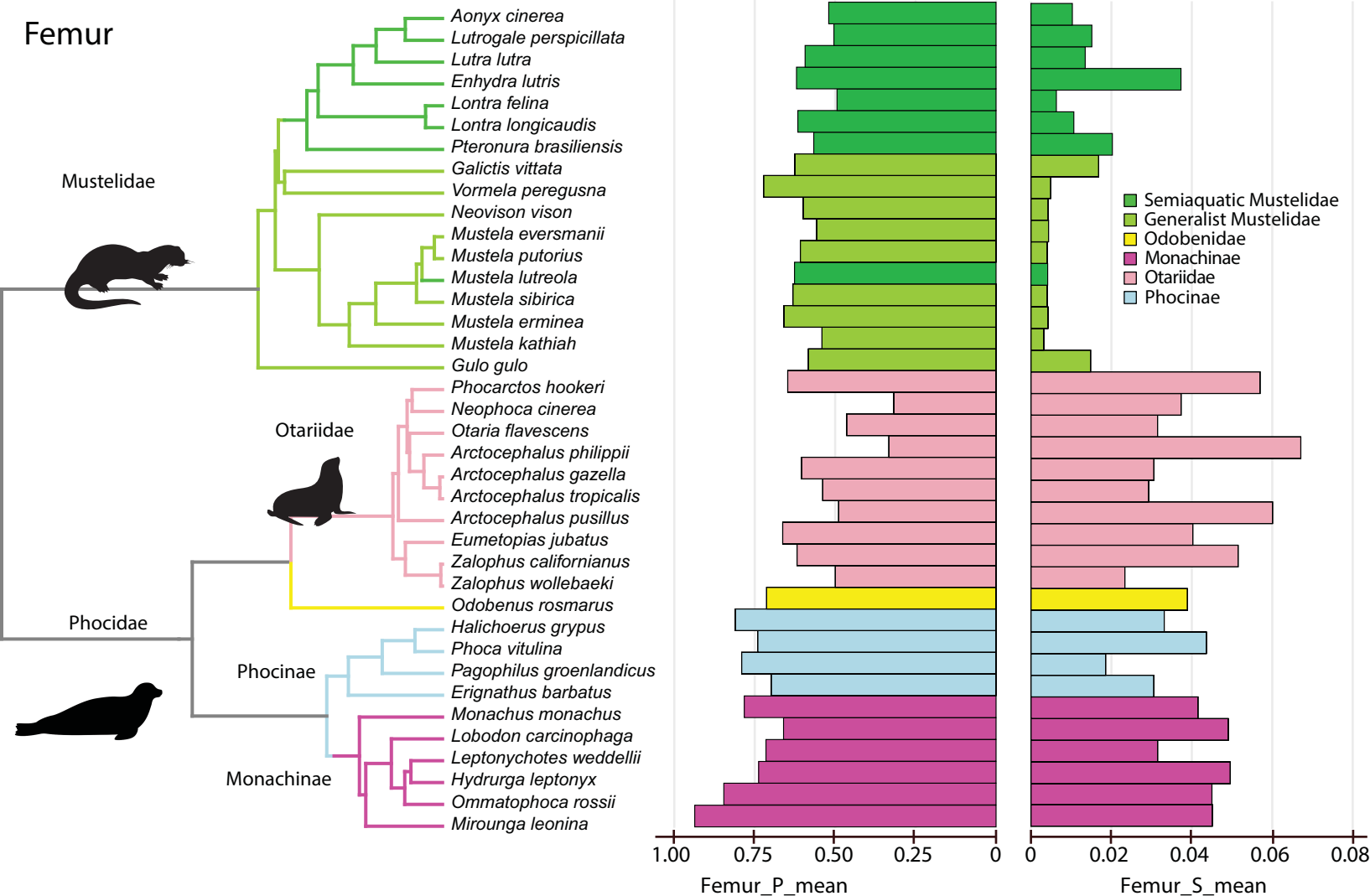

Supplement: Supplementary file 5 — FIGURE S5: Bar plots for parameters P and S in all femora sampled, colored by categories. [file AR-309-2124-s006.pdf]

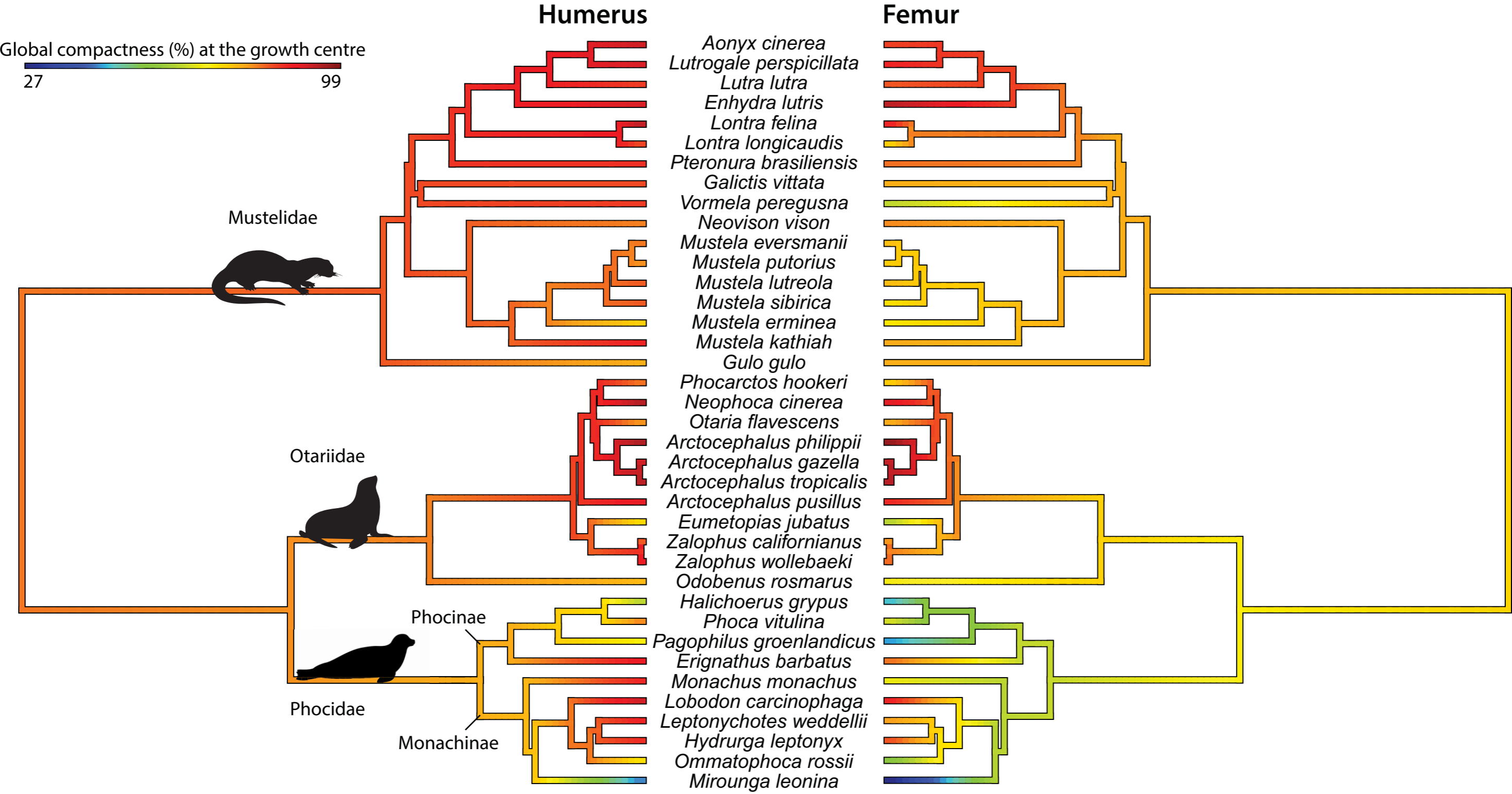

Supplement: Supplementary file 6 — FIGURE S6: Phylogenetic mapping of the global compactness (%) acquired at the growth center of the humerus (left) and the femur (right) for each species sampled (maximum likelihood estimation; contMap function, phytools package (Revell, 2012)). [file AR-309-2124-s003.pdf]

A

Humerus

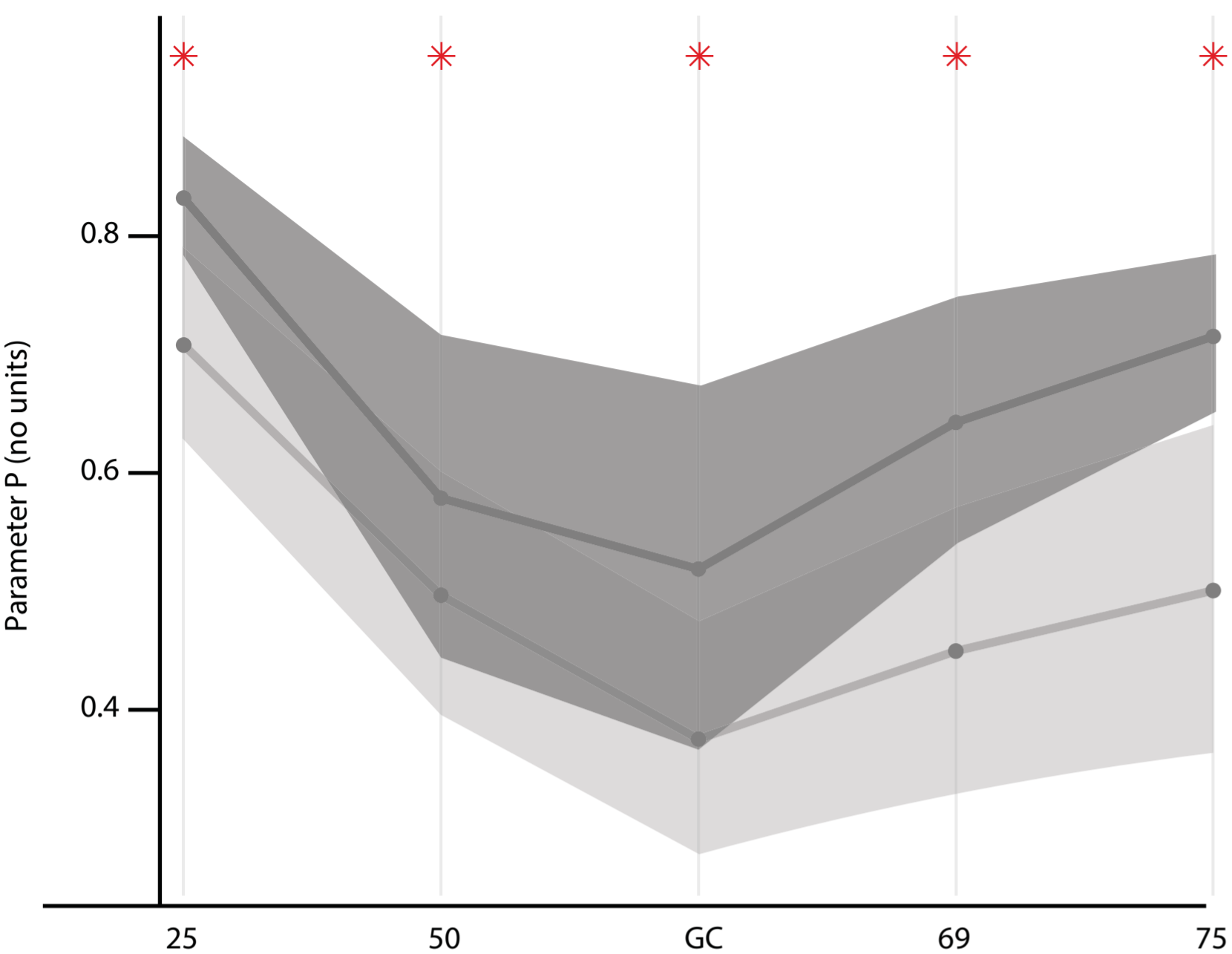

B

Femur

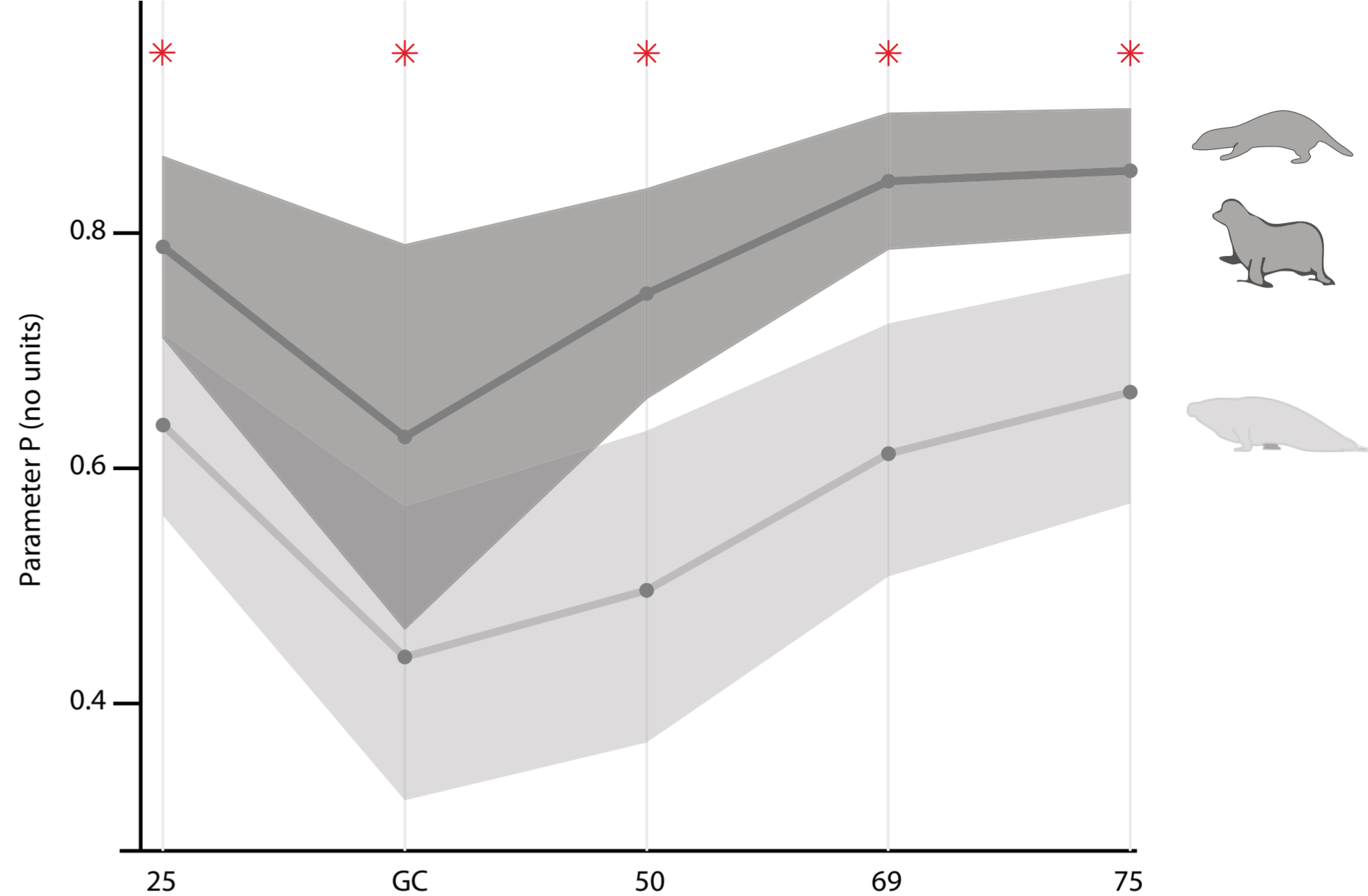

C

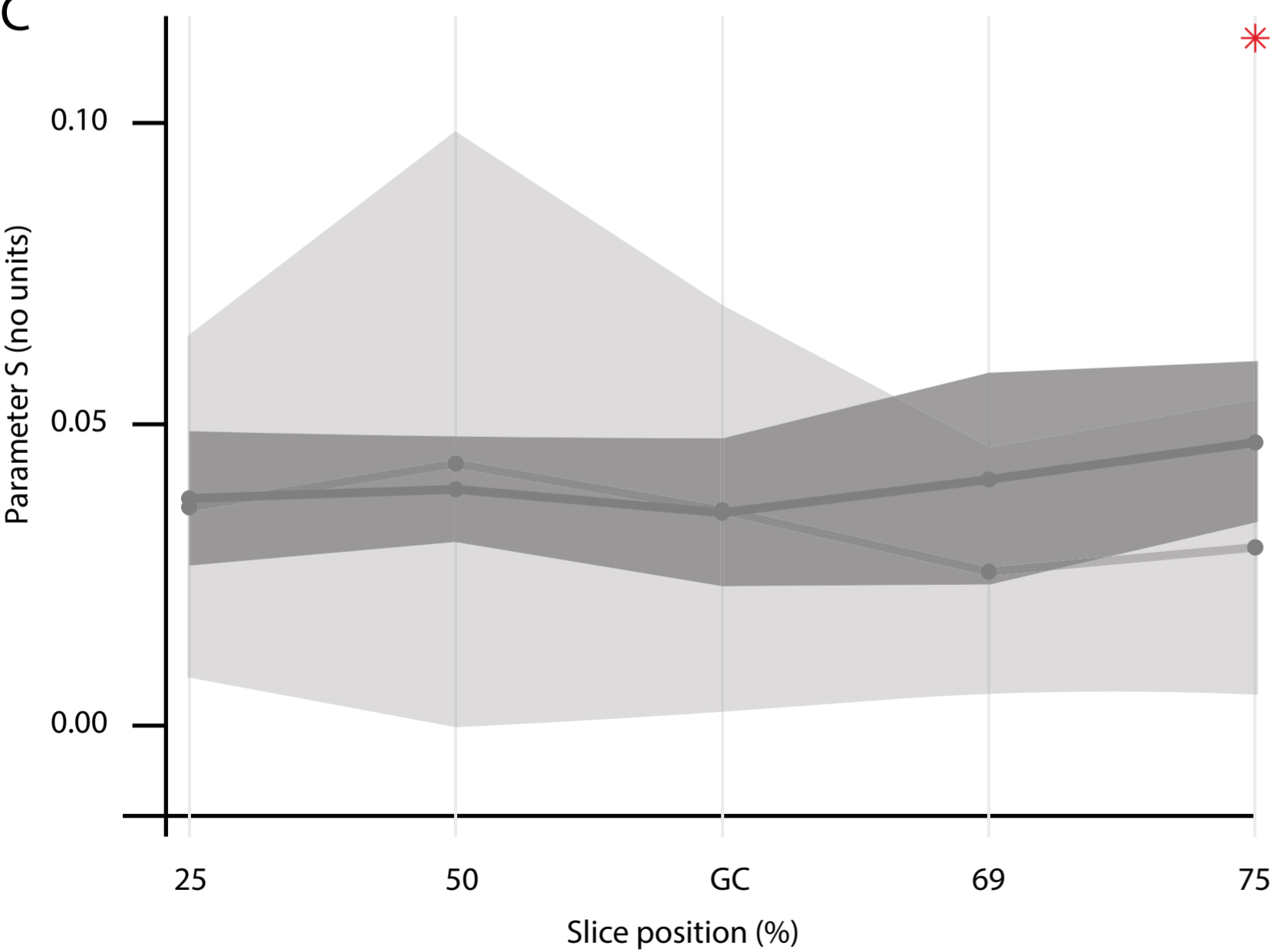

D

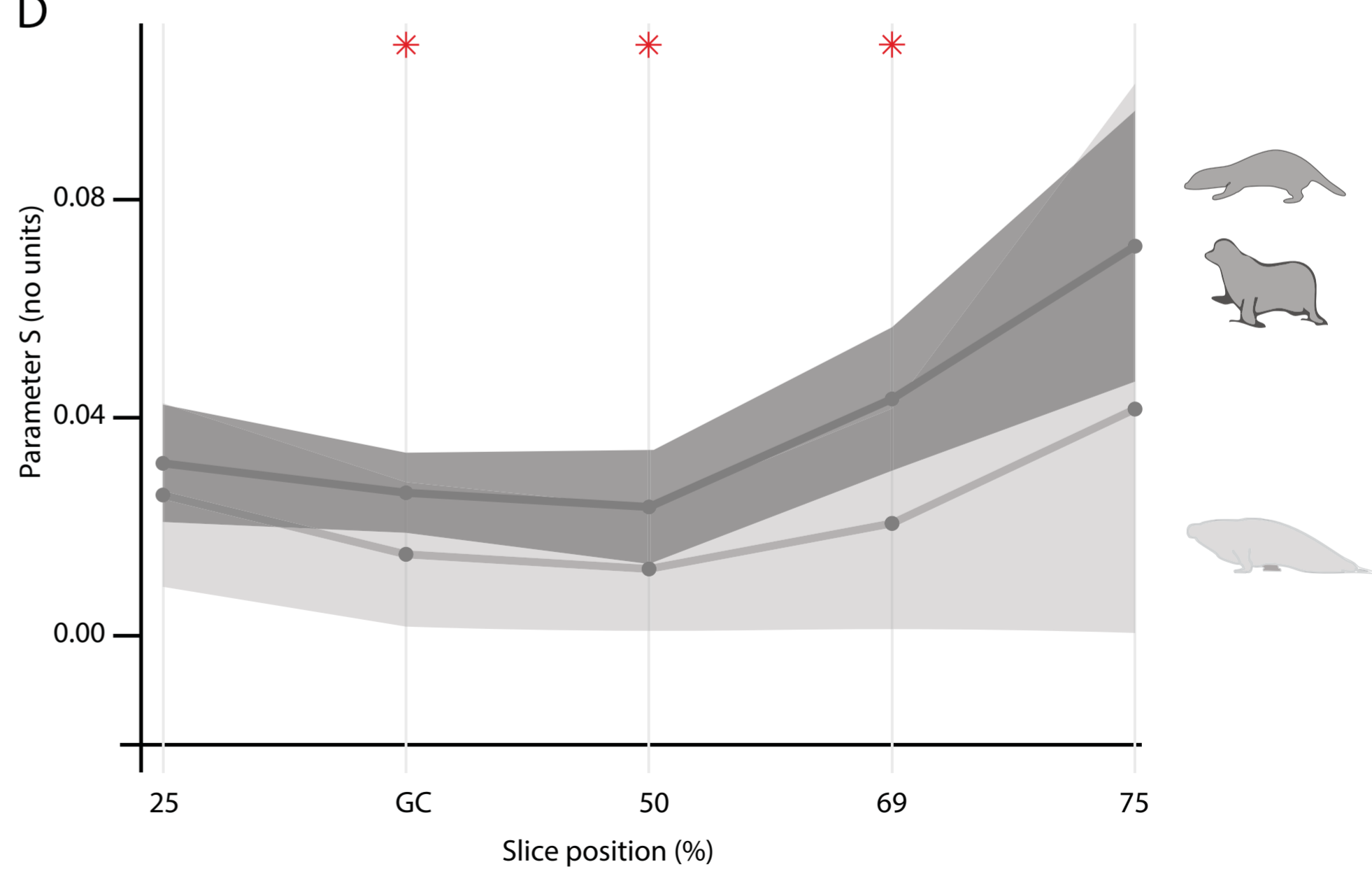

All-limb weight-bearing  
 Non-weight-bearing hind limbs

Supplement: Supplementary file 7 — FIGURE S7: Bone medullocortical transition described by parameters P and S at five positions (percentage of bone functional length; GC, growth center) along the proximo‐distal axis of the humerus (a, c) and the femur (b, d). P represents the distance of the medullocortical transition from the center of the cross‐section (a high P indicates a wide medullary region, i.e., a thin cortex). S describes the transition; a low S indicates an abrupt transition while a high S indicates a gradual one. Thick lines represent the mean value by category, and the shadow represents the variance in each group; red stars show positions where a significant difference (p‐value T–K adjusted) was found between the second‐level categories. [file AR-309-2124-s004.pdf]
